# Dynamics of Interlayer Na-Ions in Ga-Substituted Na_2_Zn_2_TeO_6_ (NZTO) Studied by Variable-Temperature Solid-State ^23^Na NMR Spectroscopy and DFT Modeling

**DOI:** 10.1021/acsphyschemau.3c00012

**Published:** 2023-05-04

**Authors:** Frida Sveen Hempel, Charlotte Martineau-Corcos, Federico Bianchini, Helmer Fjellvåg, Bjørnar Arstad

**Affiliations:** † SINTEF Industry, Forskningsveien 1, 0373 Oslo, Norway; ‡ Department of Chemistry and Center for Materials Science and Nanotechnology, University of Oslo, Oslo 0371, Norway; § CortecNet, 7 avenue du Hoggar, 91940 Les Ulis, France

**Keywords:** solid-state NMR, ^23^Na relaxation rates, Na dynamics, NZTO, layered materials, DFT AIMD

## Abstract

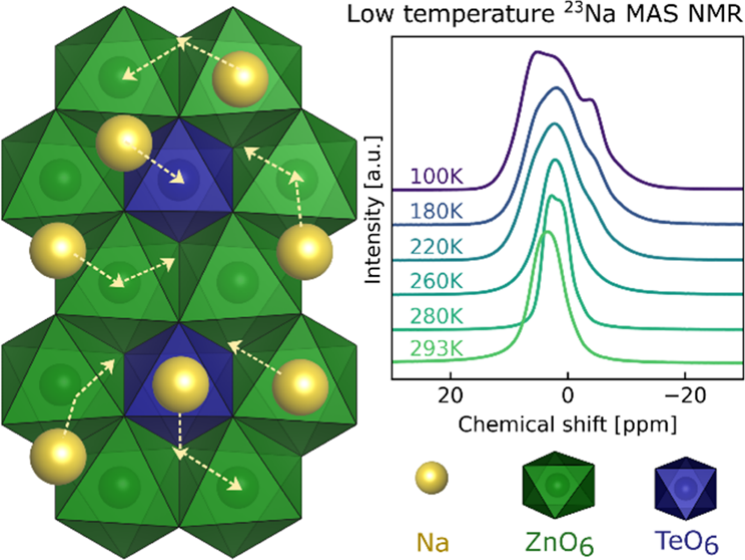

Local Na-coordination and dynamics of Na_2–*x*_Zn_2–*x*_Ga*_x_*TeO_6_; *x* = 0.00 (NZTO), 0.05, 0.10, 0.15, 0.20, were studied by variable-temperature, ^23^Na NMR methods and DFT AIMD simulations. Structure and dynamics were probed by NMR in the temperature ranges of 100–293 K in a magnetic field of 18.8 T and from 293 up to 500 K in a magnetic field of 11.7 T. Line shapes and *T*_1_ relaxation constants were analyzed. At 100 K, the otherwise dynamic Na-ions are frozen out on the NMR time scale, and a local structure characterization was performed for Na-ions at three interlayer sites. On increasing the temperature, complex peak shape coalescences occurred, and at 293 K, the Na NMR spectra showed some averaging due to Na-ion dynamics. A further increase to 500 K did not reveal any new peak shape variations until the highest temperatures, where an apparent peak splitting was observed, similar to what was observed in the 18.8 T experiments at lower temperatures. A three-site exchange model coupled with reduced quadrupolar couplings due to dynamics appear to explain these peak shape observations. The Ga substitution increases the Na-jumping rate, as proved by relaxation measurements and by a decrease in temperature for peak coalescence. The estimated activation energy for Na dynamics in the NZTO sample, from relaxation measurements, corresponds well to results from DFT AIMD simulations. Upon Ga substitution, measured activation energies are reduced, which is supported, in part, by DFT calculations. Addressing the correlated motion of Na-ions appears important for solid-state ion conductors since benefits can be gained from the decrease in activation energy upon Ga substitution, for example.

## 1 Introduction

Dynamics of ions in solids is essential for many material applications, e.g., ion batteries, membranes, and sensors. In this context, layered oxide (2D) materials are of high interest as they may intercalate ions with mobility within layers in contrast to other materials where mobility takes place between defects, often in three dimensions (3D). Modern Li-ion batteries are examples where layered oxides are used as cathode materials.^1^ Furthermore, layered oxides have also come into focus as solid-state electrolytes (SSEs). Compared to liquid electrolytes, SSEs may withstand higher potentials and improve battery properties; they may be less toxic, are more fire-resistant, and allow denser packing of battery cells, as well as making the separator redundant.^2,3^ More recently, scientific and technological development of Na-based batteries has been rapid, with certain application areas clearly evident, and the general availability of sodium implies no concern on raw material limitations.^4^ Due to the complex situation of ion dynamics in such material classes, a fundamental understanding of controlling factors and structure–dynamic relationships are important for designing improved materials with high performance.

For this purpose, nuclear magnetic resonance (NMR) spectroscopy is one of a few powerful experimental methods that may give both local structural and dynamic information. Depending on the available magnetic field, the isotope, the temperature, and type of experiment, NMR methods can probe a wide range of dynamic rates, reveal atomic and molecular details, as well as provide activation energies of the processes. While slower dynamics is visible through line shape perturbations, dynamic rates at the order of the Larmor frequency for the given nuclei at the applied field, ω_0_ (≈10^–9^ s^–1^), are best probed by spin–lattice relaxometry (SLR).^5^ The relaxation time in the laboratory frame, characterized by the time constant *T*_1_, measures the fastest dynamic processes, but slower processes can be measured by SLR in the rotating frame (*T*_1_ρ__)^6^ and by spin–spin relaxation (*T*_2_).^7^ The slowest dynamic rates are studied with 2D chemical exchange experiments or other methods like, e.g., spin alignment echo NMR.^8^ By measuring relaxation rates over a wide temperature range, the activation energy *E*_a_ and jump rates, τ^–1^, of the underlying dynamic process can be extracted.

The jump rate is assumed following an Arrhenius relationship like
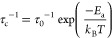
1with a pre-exponential factor in the order of phonon frequencies^9^ or the minimum correlation time at infinite temperature.^10^ The relaxation rate *T*_1_^–1^ is related to the correlation time, τ_c_, as in the following equation
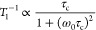
2

ω_0_ is the Larmor frequency of the nuclei in question and τ_c_ is from eq 1. Depending on the actual relaxation mechanism, there will be numerical constants multiplied with one or more expressions as in eq 2. The constants and numeric values will depend on the actual relaxation mechanisms and their contribution. A plot of log (1/*T*_1_) vs *T*(K) will, for uncorrelated 3D dynamics, with the so-called BPP behavior,^11^ be symmetrical, with the activation energy *E*_a_ for the dynamic process determined directly from the slopes. This is, however, rarely true for a real solid material, especially in the low-temperature region (ω_0_ τ_c_ ≫ 1), where dynamics is hindered, and a smaller slope is typically observed. Such correlated motion can be caused by vacancy diffusion mechanisms, structural disorder, and Coulombic interactions.^5^ The high-temperature region (ω_0_ τ_c_ ≫ 1) is frequency-independent for a 3D conductor, but systems with a reduced dimensionality for the conduction will show a reduced slope.^12,13^ Provided that the dimensionality is known, *E*_a_ can still be determined on the high-temperature slope. In order to describe in more detail ion diffusion in two-dimensional conductors, the following empirical equation has been put forward^14,15^
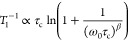
3

β expresses the frequency dependencies of the relaxation rates and is expected to be 2 for a random noncorrelated diffusion process. A lower value of β indicates correlated diffusion processes. A fit of this equation, coupled with eq 1, yields activation energies and information on ion correlations.

Before we briefly describe some relevant NMR relaxation studies for Li/Na layered materials, some word on notation is in place. Layered oxides with Na as the mobile cation can be denoted as Na*_x_*MO_2_ with 0 < *x* ≤ 1, with either one or more metal or metalloid elements M. These are classified using an *AX*-notation created by Delmas et al. based on the stacking of layers of edge-sharing MO_6_ octahedra.^16^*X* denotes the number of distinct MO*_x_*-blocks, while *A* denotes the coordination type for the Na^+^ cations, prismatic (P) or octahedral (O). The most common types are P2 and O3. Previous studies of Na dynamics in layered materials are mainly focused on cathode materials, as this has widespread use for these layered oxides. The layered Na_0.8_CoO_2_ (cathode) material undergoes an abrupt phase transition between 292 and 291 K, as seen by ^23^Na NMR relaxometry.^17^ There is no change in the narrow central transition peak but a reappearance of satellite peaks. The observation indicates a “melting” of the Na layer into a 2D-liquid state, similar to that for Na^+^-dynamics in β-alumina.^18^ The strength of the methodology is evident from studies of peak coalescence in P2- and P3-type phases of Na*_x_*CoO_2_.^19^ At high temperatures, the P2-phase peak coalesces into a single second-order quadrupolar line shape due to ion exchange. For the P3 phase, changes occur toward a Gaussian shape, as the exchange is between sites symmetric in the *ab-*plane but antisymmetric in the *c*-direction, which leads to a cancellation of the *V_zz_* contribution and suppresses the quadrupolar line shape. ^23^Na NMR of Na_2/3_Ni_1/3_Ti_2/3_O_2_ shows a relatively symmetrical peak shape at room temperature, suggesting fast Na^+^ dynamics.^20^ There is no quadrupolar line shape at lower temperature, which is suggested to be due to a dominance of magnetic anisotropy and paramagnetism over the quadrupolar interaction. In the O3-type Na_0.8_Ni_0.6_Sb_1.2_O_2_, ^23^Na NMR demonstrates off-centering of the Na-site as a response to the surrounding Na-vacancies.^21^

**Figure 1:**
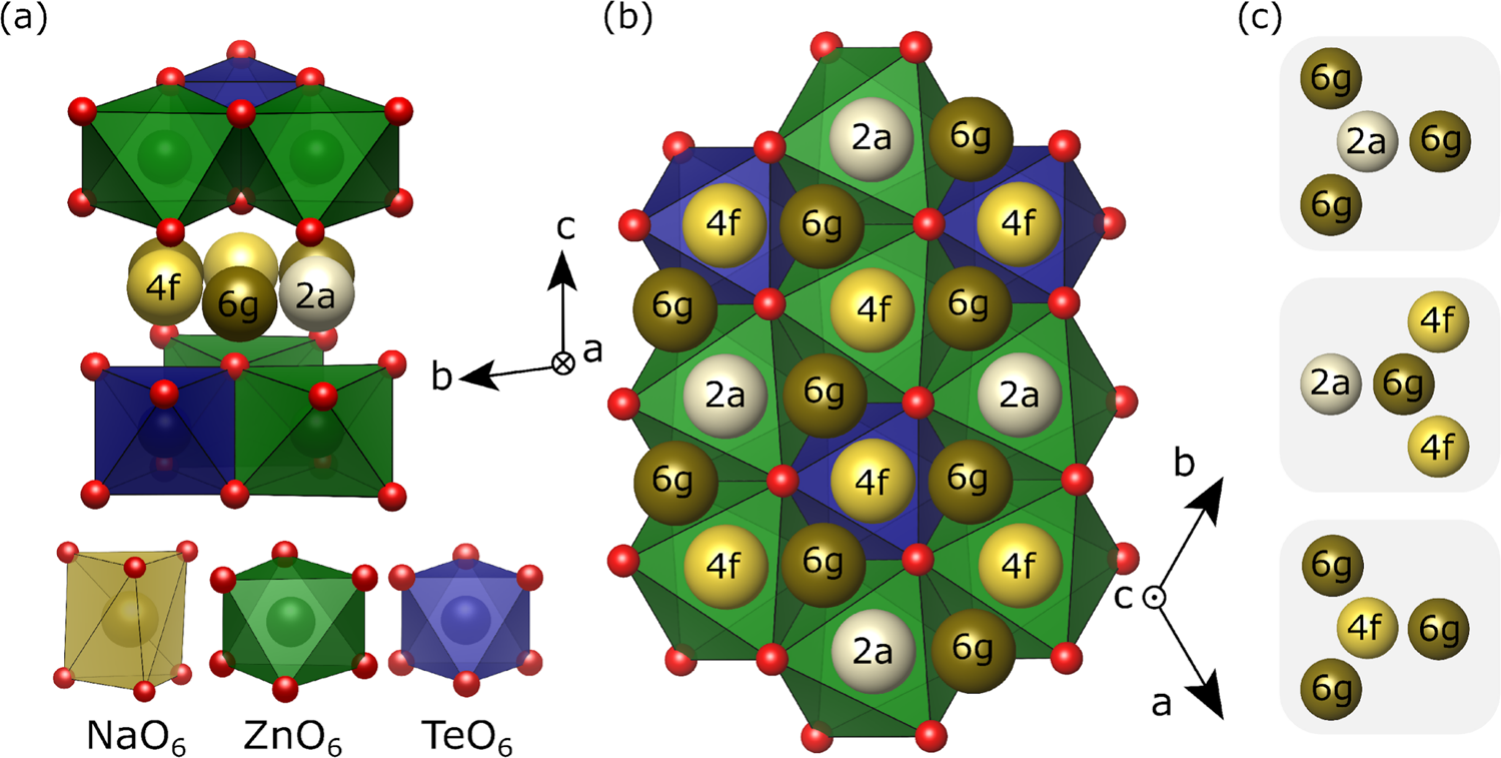
(a) Side view of the Na_2_Zn_2_TeO_6_ (NZTO) structure (P2 type; space group *P*6_3_22). (b) Coordination polyhedra of Na, Zn, and Te. The different sites in the Na layer are highlighted in color. Note that two out of six Na positions per unit cells are filled. (c) The three different Na-sites with closest neighbors. Note that the 2a and 4f positions have only 6g as the closest neighbor.

The currently investigated layered tellurates have been explored as battery materials,^22,23^ with Na_2_Zn_2_TeO_6_ (NZTO) being a candidate as an SSE.^24^ NZTO takes a P2-type structure (Figure 1; space group *P*6_3_22), with six face-sharing prisms allocating two Na-ions per formula unit. There are three nonequivalent Na-sites (2a, 4f, 6g), differentiated by the surrounding cations in the dense layers above and below. The 6g-based prisms share edges with six-framework octahedra, whereas the 2a- and 4f-based prisms are sharing faces with the octahedra. The 4f-sites are located between Te and Zn-octahedra, whereas the 2a-sites are between Zn-octahedra. Experiments show that the latter site is least favorable for Na.^22,25^ Note that we below distinguish between the ideal crystallographic sites (i.e., the 2a, 4f, or 6g-*site)* and the three different types of prismatic coordination polyhedra that are connected with these sites (2a-, 4f-, or 6g-prisms). The latter ones are typically being accompanied by a distribution of deformations. “Site” will therefore be used for crystallographic symmetry, while “prism” will be used when the chemical environment is the important aspect.

For NZTO, the Na ionic conductivity increases on substitution of Zn^2+^ with Ga^3+^, as the concomitant reduction in the Na^+^ content decreases the Na^+^–Na^+^ repulsion and increases the vacancy concentration.^26^ Introduction of Ga is suggested to decrease grain boundary resistance.^27^ Enhanced conductivity is also further reported for Ca-substitutions in the Na layer.^28^ Ca insertion increases the layer distance, which tentatively makes all Na-sites equally favorable. *Ab initio* molecular dynamics (MD) simulations of NZTO suggest a disordered Na-distribution, revealing that the honeycomb arrangement of the Zn/Te layer does not translate into Na ordering.^25^ The very similar cathode material Na_2_Ni_2_TeO_6_ (NNTO) has been investigated in a series of MD simulations, showing that disorder and ion–ion correlations influence ionic conductivity.^29^ From MD data, Sau and Kumar found that a 20% reduction of Na^+^ concentration in the interlayers significantly changed the energy landscape and Na-conductivity,^30^ mainly due to a decreased ion–ion repulsion.^31^

In this article, we present variable-temperature (100–500 K) ^23^Na solid-state (static and MAS) NMR spectroscopy data, supported by DFT modeling, to reveal the Na dynamics in NZTO and Ga-substituted derivatives. NMR experiments (at a magnetic field of 18.8 T) from 100 to 293 K were used for line shape analyses, while NMR experiments (at a field of 11.7 T) were carried out to study line shape variations and to measure *T*_1_ relaxation time constants up to about 500 K. The line shape analyses were used to reveal the onset of Na dynamics at very low temperatures (close to 100 K), and our simulations suggest a complex situation of a three-site exchange system and averaging of interactions. Simulation of the *T*_1_ measurements provided activation energies for the dynamic Na processes and revealed trends in stochastic processes influenced by Ga doping. Substitution of Ga resulted in a lower activation energy and correlated Na dynamics. The DFT modeling gave results consistent with experimental activation energies.

## 2 Experimental Section

### 2.1 Materials

Samples of Na_2–*x*_Zn_2–*x*_Ga_*x*_TeO_6_ with *x* = 0.00, 0.05, 0.10, 0.15, and 0.20 were synthesized using a conventional sol–gel synthesis, as described earlier.^32^ The procedure is reported in S1 for completeness of this work.

### 2.2 Powder X-ray diffraction (XRD)

Powder X-ray diffraction (XRD) data were measured on a Bruker D8-A25 diffractometer, using CuK_α1_ radiation, a Ge (111) Johansson monochromator, and a Lynxeye detector, for the 2θ range of 10–128°, with a step size of 0.005°. Rietveld refinements against the collected data were performed using Topas v6.^33^

### 2.3 Nuclear Magnetic Resonance (NMR)

^23^Na (*I* = 3/2) magic angle spinning (MAS) NMR single transient spectra were acquired at 11.7 T using a Bruker Avance AV III WB spectrometer equipped with a 4 mm double channel probe head at a MAS frequency of 10 kHz. The applied ^23^Na resonance frequency was 132.29 MHz, and 400 free induction decays (FIDs) were accumulated for each spectrum. Each pulse was 1.5 μs long, and we applied a recycle delay of 0.5 s. The magnetic field was adjusted by setting the high-frequency peak of adamantane to 38.48 ppm. For referencing the ^23^Na spectra, we used 1 M NaCl(aq). The chemical shift of ^23^Na(aq) was set to 0 ppm. *T*_1_ rates of ^23^Na were recorded using a saturation recovery sequence at a MAS rate of 10 kHz.

The samples are stored in Ar or in a desiccator, however, being exposed to air during handling and rotor packing. The number of adsorbed water molecules per Na atom was therefore estimated by a method described in S2. The *x* = 0.20 showed the largest water content, with one water molecule per 119 Na-ions. For *x* = 0.15, 0.10, 0.05, and 0.00, the ratios are 197, 214, 249, and 315, respectively. The estimated level of hydroxyl groups is similar to the water levels. Based on this, we assume that the ^23^Na NMR results should not be significantly influenced by the presence of water/OH-groups. Even with significant uncertainties, it is clear that the number of water molecules increases with the Ga content.

^23^Na low-temperature (LT) MAS and static NMR spectra were recorded on an 18.8 T Avance III WB using an LT-MAS 3.2 mm probe in the temperature range of RT to 100 K. The temperature was calibrated using KBr.^34^^23^Na NMR spectra are referenced to 1 M NaNO_3_(aq) at room temperature. In static conditions, a single-pulse ^23^Na spectrum was recorded. Under MAS conditions (12.5 kHz), single-pulse spectra were recorded. The pulse duration was 2.25 μs, with a recycle delay of 0.5 s and 256 FIDs accumulated per spectrum. *T*_1_ rates were recorded using a saturation recovery sequence at a MAS of 12.5 kHz. All our relaxation measurements appeared to be described very well with one exponential function. We could not distinguish any multicomponent relaxation in the spectra; hence, all data are based on the total areas of the whole multicomponent peak.

Before Fourier transform of the averaged FIDs, zero filling and apodization were applied to improve line shape definitions and signal to noise. The apodization was done by multiplying the FIDs with a decaying exponential window function with a processing line broadening (LB) factor of 250 Hz (^23^Na) and 50 Hz (^125^Te). All NMR spectra were adjusted by signal phasing and baseline corrections. Curve fitting was performed using DMfit.^35^

### 2.4 DFT Calculations

DFT simulations were performed using the Vienna Ab initio Simulation package (VASP, version 5.4.4)^36−39^ and expands our previous works,^32,40^ where configurations were used for *ab initio* molecular dynamics simulations (AIMD) to compute ionic mobility in Ga-doped NZTO. Structural optimization calculations were conducted to obtain reliable starting configurations. All computations make use of the conjugate gradient algorithm.

The Ga-doped NZTO is modeled using a 3 × 2 × 1 supercell (24 formula units, 264 atoms; 16.03 × 18.48 × 11.37 Å^3^) of the optimized configuration from our previous work,^32^ large enough to make interactions between point defects and their periodic images negligible and to perform molecular dynamics simulation.

AIMD calculations were performed for the supercell model of NZTO and for the two most stable Ga-doped configurations. Their stoichiometries are Na_48_Zn_48_Te_24_O_144_, Na_46_Zn_46_Ga_2_Te_24_O_144_, and Na_44_Zn_44_Ga_4_Te_24_O_144_. For brevity, the Ga-doped systems are labeled as 2Ga and 4Ga. The simulations were performed within both the canonical (NVT) and the microcanonical (NVE) ensembles: the former for thermalization of the system and the latter for production. In both cases, we use the same parameters as described in the Supporting Information (SI). Calculations within the canonical ensemble are modeled using the Nosé thermostat for controlling temperature oscillations.^41−43^ The Nosé mass parameter is set so that the period associated with these fluctuations is 40 fs. A time step of 1 fs was found to be sufficiently small to avoid sudden jumps in the total energy of the system during the simulation.

Production calculations, performed in the microcanonical ensemble, compute 50 ps of dynamics at four distinct temperatures: 750, 1000, 1250, and 1500 K. Diffusion was not observed in the trajectory at 500 K, and these data were therefore not relevant. The analysis of the trajectories relies on three packages: MDANSE,^44^ the atomic simulation environment (ASE),^45,46^ and QUIPPY, the python interface of QUIP.^47^ Vesta is used for rendering ball-and-stick and coordination polyhedra.^48^

## 3 Results and Discussion

### 3.1 Structure

The materials were synthesized and characterized in detail in our previous work, but the main features will be presented here for completeness.^32^ Additional information is described in the S1. All materials crystallized in the P2-type phase with the space group *P*6_3_22, in accordance with previous reports, with the *x* = 0.00 and 0.05 with a minor ZnO impurity. With increasing substitution of Ga, the *c*-axis expands and the *ab*-plane contracts. The exception here is *x* = 0.15, which was thought to be connected to Na ordering or inhomogeneous filling of the layers, which was indicated in DFT simulations. The inserted Ga-atoms were shown to be placed next to Te-atoms in stoichiometric amounts as shown by ^125^Te NMR, confirming that the materials have fully substituted Zn with Ga without secondary Ga-containing phases. We also show that the Na content surrounding Te was reduced. This confirms the Ga substitution mechanism.

### 3.2 Low-Temperature^23^Na NMR

**Figure 2:**
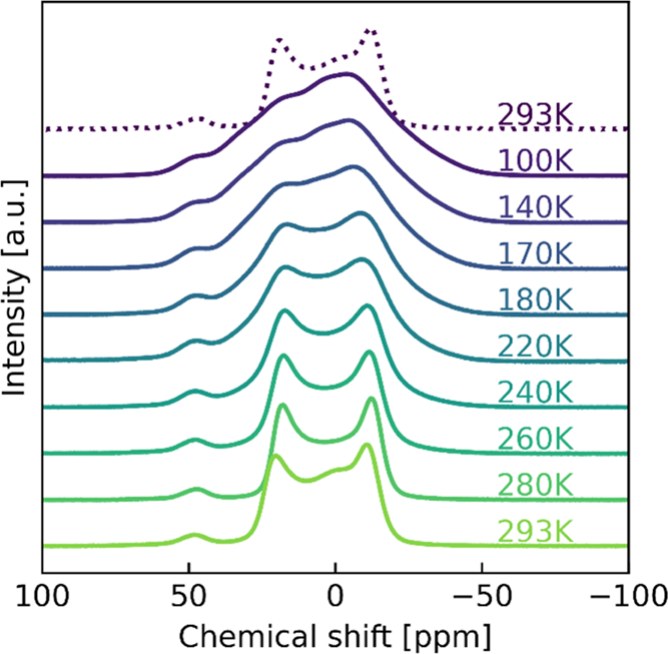
Static NMR (18.8 T) from 100 K up to 293 K. The dotted line is the 293 K peak after cooling and heating, showing the reversible change and facilitating comparison of the sample at the two end point temperatures. The feature around 50 ppm will be discussed in Section 3.4.

Line shape analyses of NZTO and its derived Ga variants were carried out from 100 K up to about 500 K to investigate Na dynamics. First, the NZTO material will be described in detail and later the Ga-doped materials will be described. *T*_1_ relaxation measurements were carried out between 293 and 500 K and will be described and analyzed after all line shape analyses. NMR spectroscopy of nonrotating samples (termed static NMR) shows all line broadening effects in a sample such as dipole–dipole interactions, quadrupole interactions, chemical shift anisotropies, and susceptibility broadenings. However, even with these seemingly complicating interactions, static NMR spectra may provide valuable information of possible dynamic processes with rates comparable to the spectrum widths as temperature is varied. At a low enough temperature, ion dynamics driven by heat will be drastically reduced and the movement of Na will “freeze out” and ^23^Na NMR spectra without time-averaged peaks can be obtained. The “freeze-out” temperature is difficult to predict; therefore, we carried out our series of ^23^Na MAS and static NMR experiments down to the lowest possible temperature that could be reached with our apparatus (100 K at 18.8 T). In Figure 2, we show a sequence of static ^23^Na spectra ranging from 100 K up to 293 K for our NZTO material.

**Figure 3:**
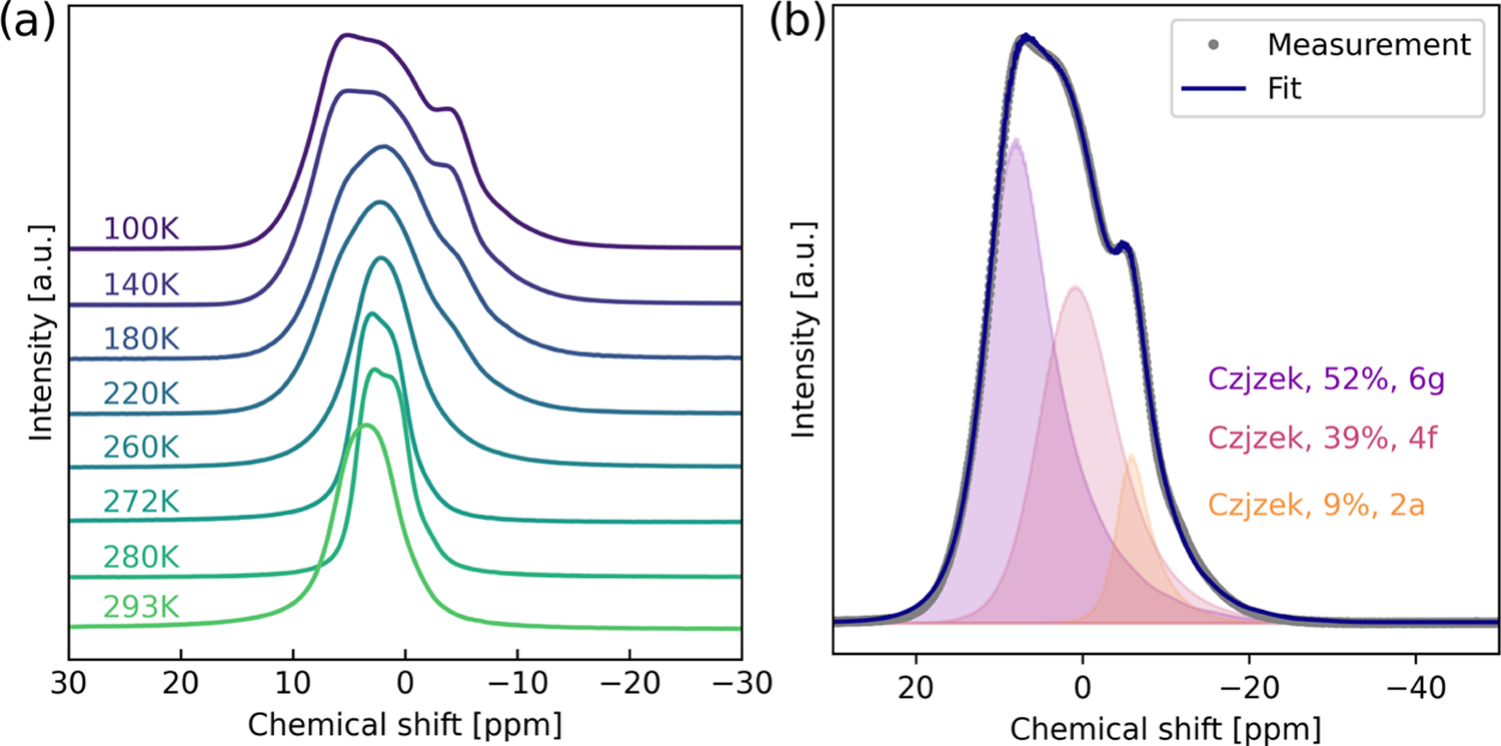
(a) ^23^Na MAS NMR spectra (18.8 T) of sample NZTO from 100 to 293 K. A plot with a larger spectral width is provided in S3. (b) The spectrum at 100 K with a decomposition shown by the filled-in distributions. All peaks are modeled using the Czjzek model as implemented in DMFit (a table is provided in S4 with details), shown with integrated intensity and the assigned Na-site, which is further discussed below.

The overall observed trend is a gradual narrowing of the spectra going from 100 K up to 293 K. Note that the peak at about 50 ppm is not changing significantly. Initially, at 100 K, the spectrum is almost a featureless shape, but with increasing temperature, a more and more distinct shape emerges. However, only a minor change between 100 and 140 K was observed; hence, it appears that for our NZTO sample, and measurement conditions (field strength, NMR method), Na dynamics is not observed. We therefore take the 100 K spectrum as our nondynamic reference state. We also note the absence of a typical quadrupolar peak shape(s) at these low temperatures. As the temperature is increased, the line shape narrows as expected if Na dynamic is present. The point of coalescence is hard to estimate but could be somewhere between 200 and 240 K. This gives a lower limit of the frequency for the dynamic process. The peak is approximately 25,000 Hz wide at 100 K, and therefore the frequency of the dynamic process at the temperature of coalescence must be at least half of this, which is 12,500 Hz. The static spectra also change between 280 and 293 K, with a shift to the left and the appearance of a central peak. At 280 K, the shape is best described as two maxima with a small component at 45 ppm. At 293 K, an extra component is visible at approximately 0 ppm. This peak is not distinguishable at lower temperatures but is visible both before and after cooling. This could suggest that the peak is a result of a dynamic process. It is safe to assume some additional broadening due to magnetic susceptibility, so the extracted values for dynamic processes from the spectra are overestimated. Due to the complicating line broadening factors and the somewhat unclear situation observed in the static experiments, we carried out a set of similar experiments as those shown in Figure 2, except with MAS, to obtain spectra with more resolved peaks. These are reported in Figure 3a. Figure 3b shows a magnified 100 K spectrum including a curve fitting into three components, their integrated areas, and assigned Na-site. Note that for peaks from dynamic systems, as in Figure 3, the chemical shifts and shapes will be a function of the relative amount and exchange rates relative to the spectral (peak region) width. Eventually, the chemical shift of a common high-temperature peak will be determined by the weighted average of the resonances when the exchange rate is much larger than the chemical shift difference (in Hz) between the peaks. Na-ions are dynamic at most of the conditions we have studied; hence, our use of the term “site” is not intended to exclude variations in positions and configurations that may be possible.

Before going further in the analysis of the temperature series for our materials, we will discuss the 100 K ^23^Na MAS NMR spectrum of the NZTO at 18.8 T in some detail (Figure 3b). At 100 K, the ^23^Na MAS NMR spectrum does not show any distinct sign of any second-order quadrupolar peak shapes but may be decomposed into three components using a Czjzek distribution,^49^ which is a model describing peak shapes originating from a distribution of quadrupolar couplings. Another possibility is a distribution of chemical shifts, with the Na not being localized to a single position. This last situation has previously been described for a similar material in the O3-form,^21^ and it is not unlikely that there should be some variance in Na positions for the P2 type. We note that the chemical shift variations between the three peaks are relatively small, which is quite reasonable considering that the difference between the three sites is only in the second coordination sphere. There are three crystallographic Na-sites (6g, 4f, 2a) with a 3:2:1 site multiplicity in NZTO, respectively. However, previously reported refinements of the Na-distribution on these sites are varied, with the 6g-site reported between 43 and 71%, the 4f-site between 22 and 55%, and the 2a-site between 2 and 12%.^22,32,40^ Na in the 2a-site is also reported to be less energetically favorable relative to Na in the 4f*-* and 6g*-*sites, both from simulations and from measurements,^22,32,40^ and is therefore likely to have the lowest occupancy. Based on this, we assign the smallest peak (9%, at ∼ −4 ppm) to Na in the 2a-site. To assign peaks to Na in 4f*-* and 6g*-* sites, we note that both the polyhedra around the 2a*-* and 4f-sites are face-sharing with two-framework layer-octahedra, while the 6g-polyhedron is edge-sharing with six-framework layer-octahedra. Therefore, we expect Na chemical shifts in 4f*-* and 2a*-*sites to be relatively similar and different from 6g. We therefore assign the middle peak (39% at ∼1 ppm) as Na in the 4f-sites and the largest peak (52% at ∼7 ppm) from Na in the 6g-sites. This is also in line with the general trend of the relative Na occupancy in these sites. We will use the term 2a-, 4f*-*, or 6g-peak for Na in these sites/polyhedra.

With the assignment of the peaks in the 100 K ^23^Na NMR spectrum, we are in a position to discuss the dynamic behavior of Na^+^ when the sample is heated. While there are some minor changes in the ^23^Na MAS spectra (Figure 3a) from 100 to 140 K, we observe that at 180 K the overall peak shape has become narrower, with a shift of the highest point to the middle peak. This change is evidence of Na dynamics between sites at these conditions. Further heating to 260 K shows an expected narrowing of the peak shape and a coalescence into practically one peak at a chemical shift of 35 ppm with an FWHM of 7 ppm. One could then expect that further heating resulted in a narrowing of this peak, but at 272 and 280 K, there are some new peak features emerging. A peak splitting appears with the highest point on the left side. At 293 K, the peak is without these features but is placed further to the left. A qualitative description of the dynamical features observed in Figure 3 may be given by considering several factors. At about 100 K, Na-ions are immobile, but from the onset of Na dynamics, the observed peak positions will be from a weighted average and the total peak shape will change significantly. Furthermore, with increasing Na dynamics dipole–dipole and quadrupolar interactions will be reduced and relaxation rates will change as well, *vide infra*. Reduction of interactions due to dynamics will lead to peak narrowing, and in addition, a reduction in the quadrupolar couplings will impose a change in the position of the peaks toward the left in the spectra. This last effect is, to some extent, already reduced at the high magnetic field strength (18.8 T) applied during these experiments compared to those at 11.7 T. The spectral features during heating from 100 K are therefore a result of changing strengths of interactions between ions experiencing three-site exchange dynamics, with some initial limitations. Our qualitative description of the observed temperature development in Figure 3 is supported by calculations of a three-site exchange model implemented in an in-house written Matlab script (see SI S5). At 100 K, Na is basically nondynamic but will, with increasing temperature, become more and more mobile. We assume that Na will eventually jump between all of the three sites 6g, 4f, and 2a. These are termed 1, 2, and 3, respectively, in the figures in S5. However, just after Na dynamics have started, but still at a low temperature, Na jump between sites should preferably go either *from* or *to* a 6g-prism^25^ and not directly between 2a and 4f. However, at higher temperatures, the rate of Na jumps may become so fast that several steps take place during acquisition; hence, for modeling purposes, jumps between all three sites must be considered. The simulations show that the observed spectral trend when going up from 100 K is better represented if there is an exchange between sites 6g and 2a in contrast to 6g and 4f, or 4f and 2a (see S5 panel 1, 2, 2X, and 2Z) and is in accordance with the above-mentioned jump order. Furthermore, to mimic the general observed peak shapes, we must include jumps between sites 6g and 2a (panel 3 in S5). To approach the trend toward the 260 K spectrum, jumps between sites 4f and 2a are included as shown in panels 4 and 5. With a further increase in jump rates for all three paths, only a peak narrowing is observed, which contrasts with what is observed at 272 K where a ″peak splitting″ with the highest intensity on the left part of the total peak shape is observed. Such an observation may originate from a significantly reduced quadrupolar coupling of one or two components relative to the other(s), and to represent this, we adjusted slightly the peak positions toward the left when going from panel 5 to 6. However, to mimic the observed trend, we also had to narrow the peak from the 6g position and adjust positions (see panels 7 and 8). Panels 7–9 are all somewhat similar as the observations around 272–280 K. Finally, by increasing all three jump rates to an equally high number and with a slight and equally reduced line broadening of all of the peaks, we were able to progress to a total peak shape that looks like the one observed at 295 K via the split peak (280 K, panel 9). In conclusion, the observed spectra are all a result of a complex interplay and influence by coupling interactions, relaxation times, relative amounts of Na at sites and to available jump-pathways (primarily at low temperature).

As a last comment on the spectra shown in Figures 2 and 3, one may note that the total peak width ratios of the static over MAS spectra are for 100 K about 4 and at 293 K almost 6. This indicates that the dynamic situation at 293 K helps to reduce the peak width in contrast to the dynamic situation at 100 K. For quadrupolar nuclei, MAS can reduce the line width by about a factor of 4.

### 3.3 Room-Temperature^23^Na NMR on NZTO

**Figure 4:**
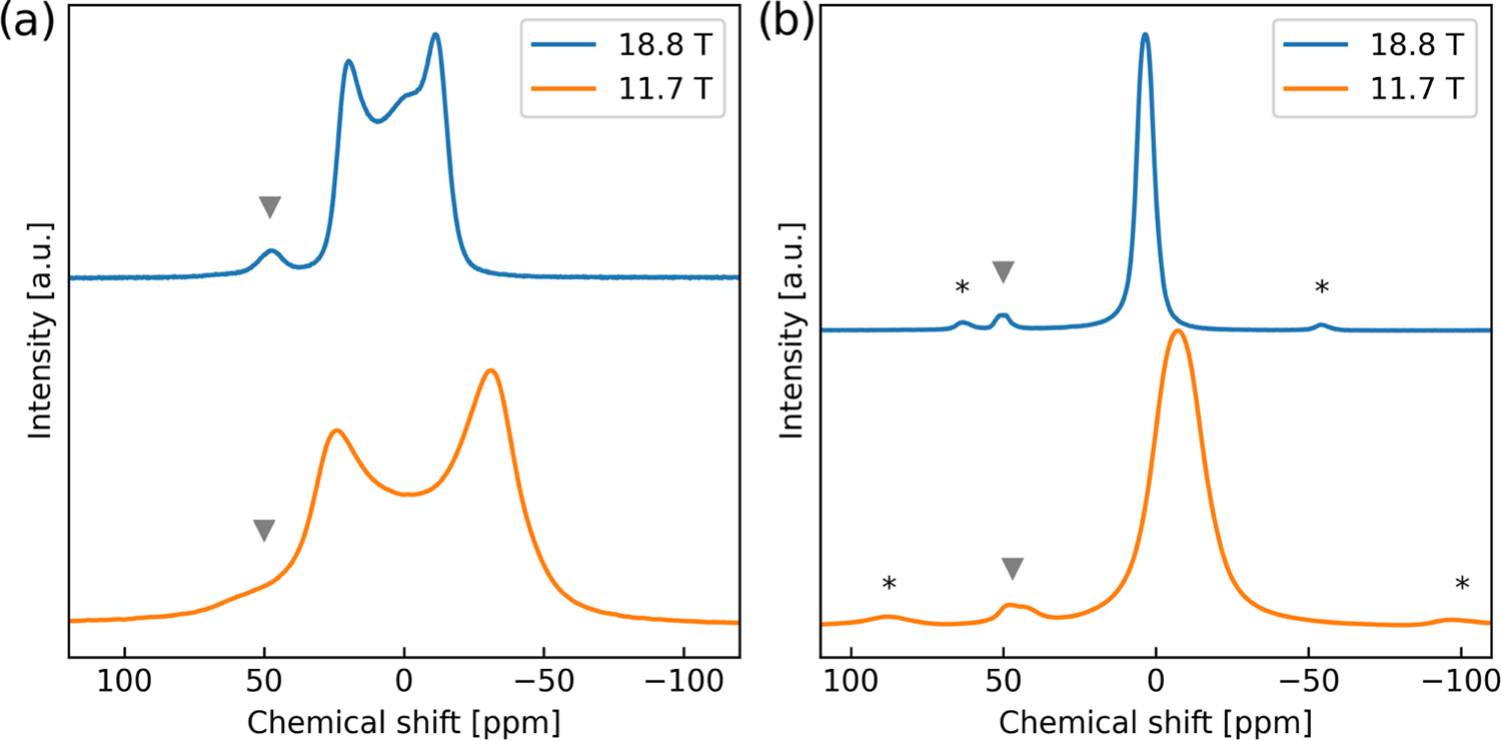
(a) Comparison of ^23^Na NMR spectra of static NZTO at magnetic fields of 18.8 and 11.74 T. (b) ^23^Na MAS NMR of NZTO at 18.8 T (MAS rate 12.5 kHz) and at 11.75 T (MAS rate 10 kHz). The feature at 50/45 ppm is denoted with a gray triangle, with spinning sidebands with an asterisk*.

Our low- (100 K) to high-temperature (500 K) ^23^Na NMR experiments have been carried out using two different magnetic fields, 18.8 and 11.7 T, respectively, with the room-temperature (∼293 K) measurement being the point of overlap. Figure 4 shows the four NZTO spectra at room temperature to be compared, two at static conditions (left) and two during MAS (right) conditions.

Overall, peaks in the spectra acquired at a higher field are narrower and slightly shifted to the left. In the MAS spectra, the main peak changes from −7 ppm at 11.7 T to 3 ppm at 18.8 T. This implies that there are quadrupolar couplings that must be described by the second-order term in the perturbative development of the Hamiltonian, as this term is inversely proportional to the magnetic field and contains a term giving rise to a shift in peak position. We know that there are Na dynamics at room temperature; hence, the effects we observe in Figure 4 are not purely due to the magnetic field variations. However, the fact that we see the effect of field variation indicates that the dynamic situation at 293 K does not have rates high enough to completely average the quadrupolar couplings at 11.7 T. One could loosely claim we have residual quadrupolar couplings at 293 K for the lowest field strength. Furthermore, the MAS spectrum recorded at 11.7 T is not fully symmetric, but at 18.8 T, the main peak is basically symmetric as seen by curve fitting with a Gaussian and/or a Lorentzian function (S6, Figure S2). The symmetry is indeed high, but the fact that two different types of functions must be used is also a sign that the situation is still somewhat complicated by various interactions. The small components at 45/5 ppm do also shift somewhat when going to the higher field. The left component does barely shift (2.5 ppm), but the right component has a somewhat larger shift (6 ppm).

### 3.4 High-Temperature^23^Na NMR on NZTO

**Figure 5:**
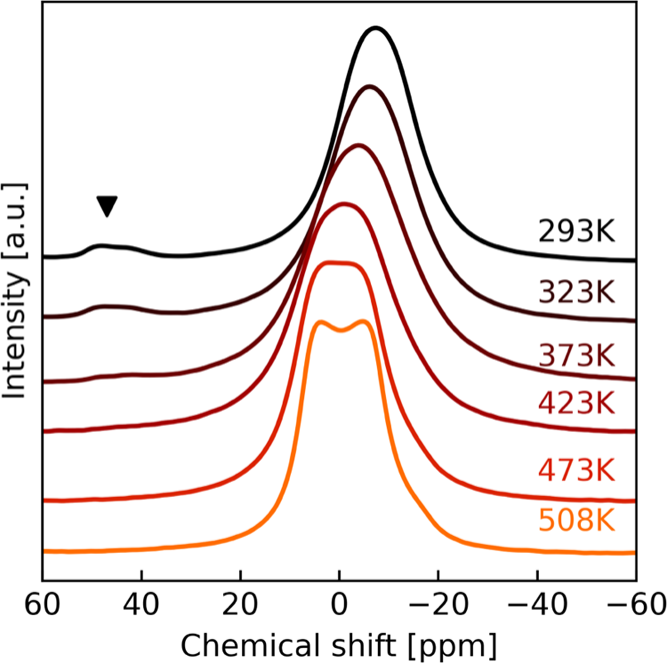
^23^Na MAS NMR (11.7 T) of NZTO heated up from 293 K (RT) to 508 K. The triangle marks the small components that reversibly merge with the main peaks at an elevated temperature.

The NZTO and variants (*vide infra*) were studied up to about 500 K at a magnetic field of 11.7 T to unravel Na dynamics through line shape analyses and via *T*_1_ relaxation constant measurements. Note that for NZTO, no phase transition has been reported until between 573 and 673 K where a gradual, reversible change has been observed in the Na-distribution, going from an orthorhombic superstructure to a hexagonal.^22,40^ The line shape variations of NZTO will be described first. Figure 5 shows a stacked plot of ^23^Na MAS spectra from 293 K up to 508 K.

Up to 423 K, the main peak keeps its shape but moves slightly to the left and the small peaks at 45/50 ppm appear to vanish. Upon cooling to 293 K after heating to 508 K, the spectrum regains its original peaks and shapes proving the reversibility of the temperature effects. Na-ions are in structural parts linked with each other and with the interlayer regions. The shift to the left (i.e., higher frequencies) may be explained by a quadrupolar interaction that is weakened by Na dynamics. NMR spectra at 473 and 508 K reveal that the peak has diverged into something that looks like two components with some intensity at the right foot of the peak at 508 K. From the line shape discussion above, the spectral features seen here must be due to variations of rates for the three-site exchange coupled with reduced interaction strengths. The exchange rates are high; hence, the peak splits must mainly be due to reduced quadrupolar and dipole–dipole interactions. Another issue that also may take place is that previous simulations indicate that the Na-distribution in one layer influences the allowed positions in adjacent layers, suggesting that the layers are not as independent as previously assumed and a more complex situation may be expected.^32^

### 3.5 NMR on Ga-Substituted NZTO from 100 to 293 K

**Figure 6:**
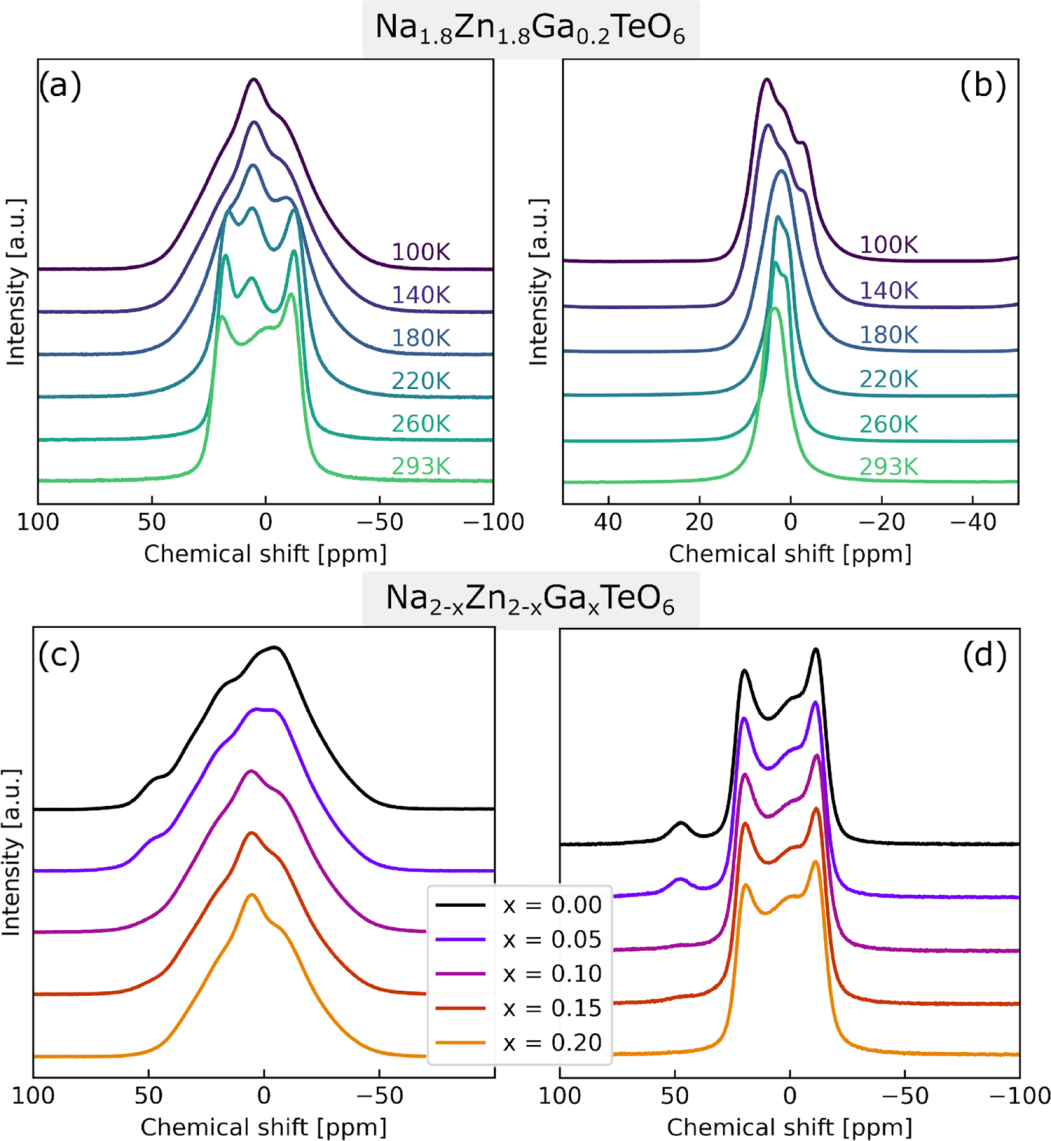
(a) Static and (b) MAS at a rate of 12.5 kHz, and ^23^Na NMR spectra of Na_2–*x*_Zn_2–*x*_Ga_*x*_TeO_6_ (*x* = 0.20) in the temperature range of 100–293 K (18.8 T). Static ^23^Na NMR spectra (18.8 T) of Na_2–*x*_Zn_2–*x*_Ga_*x*_TeO_6_ (*x* = 0.00, 0.05, 0.10, 0.15, 0.20): (c) 100 K and (d) 293 K.

With a basic description and understanding of the NZTO material from 100 K up to 508 K, we continue with NMR data analyses for the Ga-doped samples, presenting figures describing the most characteristic trends. Figure 6 shows a panel of selected static and MAS ^23^Na NMR spectra, between 100 and 293 K, all at 18.8 T, of Na_2–*x*_Zn_2–*x*_Ga_*x*_TeO_6_ (*x* = 0.00, 0.05, 0.10, 0.15, 0.20). Additional static and MAS spectra of Ga-substituted samples are reported in S7 Figures S3 and S4.

In the top row, ^23^Na NMR data of the sample with the highest Ga doping (*x* = 0.20) from 100 K up to 293 K is shown: (a) static and (b) MAS experiments. The bottom row shows static ^23^Na NMR spectra (18.8 T) of Na_2–*x*_Zn_2–*x*_Ga_*x*_TeO_6_ (*x* = 0.00, 0.05, 0.10, 0.15, 0.20): (c) 100 K and (d) 293 K. The spectra in Figure 6a show a clear coalescence of peaks with increasing temperature. A similar coalescence is also shown for intermediate compositions. At 220 K, the peak width in Figure 6b appears to have reached its minimum, in contrast to NZTO where it can be estimated that the minimum peak width was reached at 260 K. This is proof of higher Na dynamic rates in the Ga *x* = 0.20 sample compared to NZTO. A feature in Figure 6a is the ″middle″ peak that is present between the two outermost peaks. The NZTO sample did not show any ″middle″ peak until the spectrum acquired at 293 K. This middle peak is for most of the spectra in Figure 6a at the same position as the highest peak at 100 K until 293 K where it has changed to the right and appears broader. These observations are again indications of higher Na dynamic rates in the Ga *x* = 0.20 sample and that some exchange effects that broadens the peak are seen at 293 K.

Static spectra of the various Ga-doped samples show significant changes with increasing Ga content. The small component at around 50 ppm becomes smaller with increasing Ga content and appears to vanish for the *x* = 0.20 sample. The widths of the peak shapes are, however, very similar throughout the series. The MAS spectra of the same set at 100 K, Figure S3a, show a gradual change between components with increasing Ga content, while the 293 K spectra of the same set, Figure S3b, are more similar.

### 3.6 High-Temperature NMR on Ga-Substituted Samples

**Figure 7:**
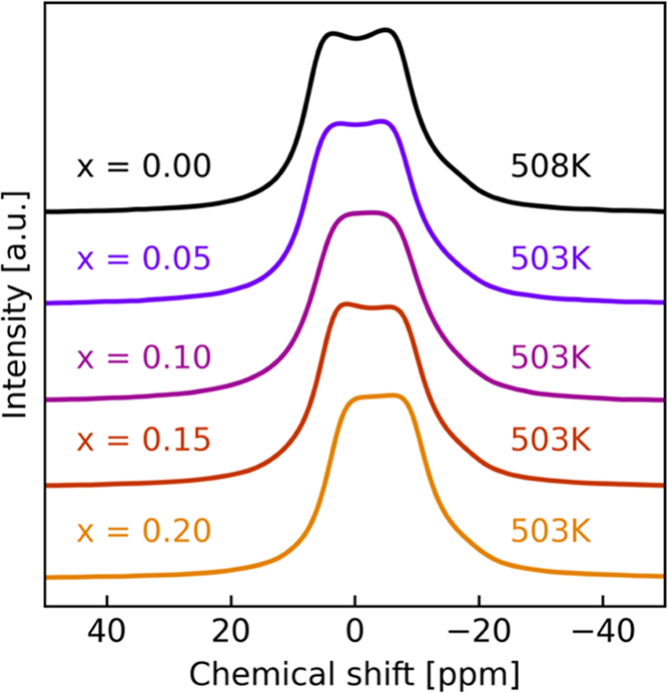
High-temperature measurement of all samples. *x* = 0.00 is measured at 508 K, while all others are measured at 503 K.

The high-temperature (RT up to about 500 K) spectral trends of all of the five samples are relatively similar, and only spectra collected at the highest temperature are shown here in Figure 7. All samples display a splitting behavior at higher temperatures, which is probably due to the above-mentioned complex three-site exchange situation of the Na-ions and reduced quadrupolar and dipole–dipole interactions.

### 3.7 Spin–Lattice (*T*_1_) Relaxation Measurements

**Figure 8:**
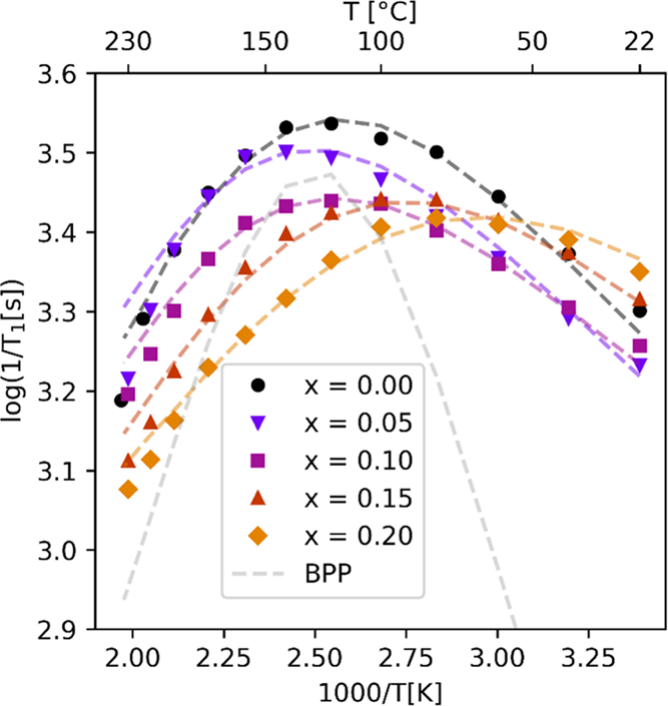
Temperature dependence for ^23^Na spin–lattice rates (1/*T*_1_[*s*]) for Na_2–*x*_Zn_2–*x*_Ga_*x*_TeO_6_ (*x* = 0.00, 0.05, 0.10, 0.15, 0.20) samples. The reduced slopes on the high-temperature side (left of maxima) are connected to a 2D dimensional process for Na-ions between layers, while the slope reduction on the low-temperature side (right of maxima) is connected to the correlated movement of ions. The dotted lines are results from modeling using eqs 1–3. The dotted, gray, symmetric curve is the expected shape of a random 3D isotropic process with an *E*_a_ of 0.32 eV, with a so-called BPP behavior.

Measurements of *T*_1_ relaxation constants were carried out to investigate dynamic trends in NZTO and how these change with the introduction of Ga in the temperature interval from 293 K up to about 500 K. These measurements probe dynamics faster than observed during peak shape variations at low temperatures, as discussed above. Deviations from a symmetric plot of log (1/*T*_1_) vs inverse temperature, as seen in fast stochastic 3-dimensional diffusion with only a simple exponential dependency of the correlation time, should be expected since Na is confined between oxide layers. Our relaxation data are plotted as log (1/*T*_1_[*s*]) vs inverse temperature (1000/*T*[K]) and shown in Figure 8. More details are provided in S8 where Table S2 lists all of the actual measured averaged *T*_1_ values used for Figure 8, and Figure S5 shows a plot of *T*_1_ relaxation data from 100 K up to 293 K, all measured at 18.8 T. Included in Figure 8 are also results (shown as dotted lines) from modeling using eqs 1 and 3 (termed as a 2D model) and a symmetric BPP curve.

All of the curves go through a maximum where the correlation time τ_c_ is equal to the inverse Larmor frequency. Hence, for each material, there is a different temperature where Na-ions have the same rates for the dynamic processes. Except for sample *x* = 0.05, the Ga-doped samples have their peak maxima shifted to lower temperatures compared to NZTO. This also indicates that Ga substitution leads to higher ion mobility and a lower activation energy for the dynamic process compared to NZTO, which is also a conclusion from the line shape analyses at low temperatures described above. Furthermore, for NZTO, it appears that a rather steady slope is obtained at the highest temperatures and that the *x* = 0.05, 0.10, and 0.15 samples seem to converge toward this approximate slope. The *x* = 0.20 sample shows a clear shift in its slopes. In the simplest model for *T*_1_ variation with temperature, the peak shape would be symmetrical, with the slope of each side determined by the activation energy, *E*_a_. However, any correlated movement will reduce the slope of the low-temperature region, and any dimensionality in the dynamic process (ion diffusion) will reduce the slope of the high-temperature region.^7^ At sufficiently high temperatures, the activation energy for the process may be estimated using an Arrhenius relation based on data points from the highest temperatures or estimated more precisely using a suited 2D model. For a set of samples with a systematic variation, as in our case where the Ga content is adjusted, it is possible to extract trends from relaxation data even if the absolute values contain uncertainties. In Table 1, we report calculated *E*_a_ values based on a simple Arrhenius relation and from a more detailed modeling (using the 2D model mentioned above). The temperature where the correlation time is the inverse of the Larmor frequency of ^23^Na at 11.7 T (τ_c_ = 1/132.29 MHz) is reported in the last column.

**Table 1:** *E*_a_ for Na_2–*x*_Zn_2–*x*_Ga_*x*_TeO_6_ (*x* = 0.00, 0.05, 0.10, 0.15, 0.20) Samples Calculated from the Leftmost Points in the Graphs (Two Values Are Given for *x* = 0.00 and 0.05) and the 2D Model and the Temperature for Curve Maxima^a^

	*E*_a_ (eV)	*E*_a_ (eV)	
	Arrhenius relation	2D model	
*x*	(points used)	(β value in eq 3)	temperature for curve maxima, K
0.00	0.34/0.25 (2/3)	0.32 (1.30)	393
0.05	0.28/0.25 (2/3)	0.32 (1.29)	413
0.10	0.17 (3)	0.30 (1.25)	393
0.15	0.17 (4)	0.26 (1.28)	373
0.20	0.14 (4)	0.20 (1.35)	353

a The uncertainty in the 2D model *E*_a_’s is approximately ± 0.01 eV.

Both models give similar values for *E*_a_ for NZTO, which are similar to results from ion conductivity^26^ and calculations (0.33 eV, *vide infra*). With increasing Ga content, both models give reduced *E*_a_, but significantly less reduction is seen for the 2D model. The β values indicate that Na dynamics are strongly correlated. A simple model for correlated motion is when one ion blocks the jump of another, but it appears that the correlations are rather similar even with a reduced Na content. Altogether, the measured decrease in activation energy is likely to be a real effect and will be discussed more in relation to the DFT results in Section 3.8.

### 3.8 *Ab Initio* MD Simulations for Ionic Conductivity

**Figure 9:**
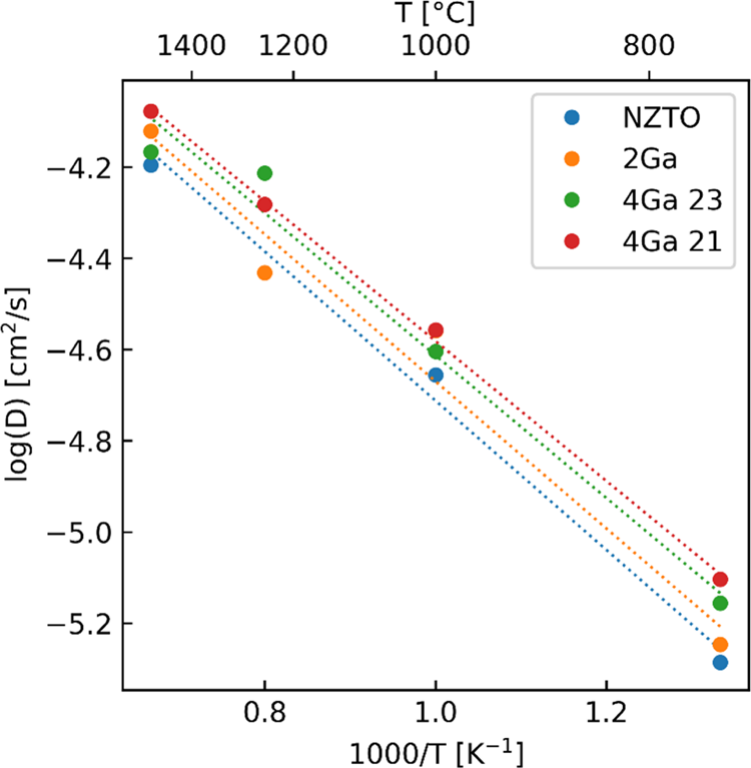
Arrhenius plot for all simulations, with the two individual 4Ga split into two distinct layers.

Three compositions of Na_2–*x*_Zn_2–*x*_Ga*_x_*TeO_6_ type with *x* = 0 (NZTO), 0.083 (2Ga), and 0.167 (4Ga) were explored with the ab initio MD simulation, the latter two, respectively, with 2 and 4Ga^3+^ ions in a supercell with 24 formula units. The construction of the model and structural insights are reported in our previous work.^32^ For each composition *x*, we consider four simulation temperatures: 750, 1000, 1250, and 1500 K. From the AIMD trajectories, it is also possible to compute the mean square displacement and, thus, the ionic mobility, as described in S9. The diffusion coefficients (D) increase slightly with higher temperatures, but the three cases exhibit very similar values at different temperatures. One thing to note is that the 4Ga structure shows clear indications of inhomogeneous Na-distribution across layers, which is distinctly more favorable than the homogeneous. The 4Ga is calculated twice for better statistics, with the two layers being reported separately. Figure 9 shows the relationship of ln(D) vs 1000/K.

The calculated activation energy for NZTO is 0.33 eV, in line with the measured range of 0.25–0.34 eV as presented above from the SLR analysis. This suggests that, even with some uncertainty, the approximate correct slope of NZTO reported above is reached. As this translates to an ionic mobility of 1.8 10^–4^ S/cm, it confirms the reported experimental range for NZTO of ∼10^–4^ – 10^–3^ S/cm.^24,26,27^ The simulations of the Ga-substituted materials have a very small decrease in activation energy, with 0.32, 0.31, and 0.30 eV for 2Ga and the two 4Ga layers, respectively, which is quite close to the values given from the 2D model for the most similar materials, i.e., those with *x* = 0.05 and 0.15. The calculated difference between the structures is within the margin of error and should therefore be considered carefully, but they do suggest a decrease in activation energy with increased Ga content, as expected. Table 2 summarizes the findings from the DFT AIMD calculations.

**Table 2:** AIMD Calculation of Na Diffusion in an Average Lattice^a^

system	act. energy [eV]	diff coeff. 10^–9^ [cm^2^/s]	mobility 10^–4^ [S/cm]
NZTO	0.33 ± 0.03	2.0	1.8
2GA	0.33 ± 0.04	3.7	3.3
4GA (23 Na)	0.31 ± 0.03	5.5	4.8
4GA (21 Na)	0.30 ± 0.03	6.9	5.6

a Diffusion coefficient and mobility are calculated at room temperature.

A discussion of the estimation of *E*_a_, ion conductivity, and the amount of Na is in place at this stage. Previous simulations have shown that in a similar material the Na_2_Ni_2_TeO_6_ ionic conductivity can be increased either by underloading the Na layer by 20%^30^ or by decreasing the Na–Na repulsion in the simulations.^31^ Both of these situations were generated by altering the physical properties or breaking electroneutrality. For a real material, the suggested route for achieving this was a reduction of the Na content, as we have done when making our Ga-substituted materials. The DFT AIMD-calculated activation energies correspond quite well with estimates from impedance spectroscopy, which shows that samples with Ga substitution *x* = 0.05–0.15 have activation energies in the range of 0.30–0.27 eV.^26^ Since our DFT-calculated activation energy is based on the mean square displacement, both of these methods provide values related to long-range movement, whereas SLR relaxation rates probe Na-ion dynamics on a shorter range. It is interesting that nudged elastic band (NEB) calculations of Na jumps in an empty Na-lattice between the framework layers in NZTO gave an activation energy of 0.09 eV^24^ and could suggest that increasing the Ga content leads to a situation where more and more Na behaves like Na in an empty lattice. It appears that the decrease in activation energy for Na jumps in the Ga samples cannot be exploited fully for increased ionic conductivity since the previously measured ionic conductivity of *x* = 0.0 and 0.2 is rather similar. Furthermore, our analysis points to a situation where an optimum composition for ion conductivity for a certain class of layered materials exists, which is what Li et al. observed.^24^ If layered material′s structure–dynamic relationships can be controlled through preparation, an important step toward creating better solid-state electrolytes can be taken.

## 4 Conclusions

We have characterized Na-coordination and dynamics in NZTO and its Ga-substituted derivatives (Na_2–*x*_Zn_2–*x*_Ga*_x_*TeO_6_ with *x* = 0.00, 0.05, 0.10, 0.15, 0.20) by variable-temperature ^23^Na NMR methods and DFT AIMD simulations. At 100 K, the Na-ions were frozen on the NMR time scale, and a structural characterization was performed. Three spectral components were assigned to the three 2a, 4f, and 6g Na-prisms, as the integrated intensities correspond well with the expected site multiplicity. Variable-temperature measurements from 100 K and upwards on NZTO showed a complex peak shape coalescence. These measurements also show that the Na spectrum acquired at 293 K had some averaging in it due to Na-ion dynamics. A three-site exchange model coupled with reduced quadrupolar and dipole–dipole couplings due to dynamics seem to explain the peak shape observations. A further temperature increase to 500 K did not reveal any new peak shape variations until the highest level, where an apparent peak splitting was observed again. Measurements of *T*_1_ relaxation time constants gave insight into Na dynamics and activation energies. Ga substitution decreased the temperatures for peak coalescence and lead to reduced activation energies for the dynamic processes of Na. The estimated activation energy for Na dynamics in NZTO from relaxation measurements corresponds well with results from DFT AIMD simulations. On Ga substitution, measured activation energies are reduced and are supported by DFT calculations. We suggest that addressing the correlated motion of Na is important for creating a better Na conductor for SSE, as the decrease in activation energy from Ga substitution can be exploited.

## Supporting Information Available

A file containing details on synthesis, water estimation by NMR, enlarged NMR spectra NZTO, simulation parameters for the Czjzek distribution, simulations of a three-site exchange system, line fitting of MAS spectrum at 18.8 T, stacked plots of Ga-doped materials, details on relaxation measurements, and details on DFT calculation of Na mobility (PDF)

## References

[ref1] Goodenough J. B.; Mizushima K.; Takeda T. Solid-Solution Oxides for Storage-Battery Electrodes. Jpn. J. Appl. Phys. 1980, 19, 305–313. 10.7567/JJAPS.19S3.305.

[ref2] Eshetu G. G.; Grugeon S.; Laruelle S.; Boyanov S.; Lecocq A.; Bertrand J. P.; Marlair G. In-Depth Safety-Focused Analysis of Solvents Used in Electrolytes for Large Scale Lithium Ion Batteries. Phys. Chem. Chem. Phys. 2013, 15, 9145–9155. 10.1039/c3cp51315g. 23649367

[ref3] Sun P.; Bisschop R.; Niu H.; Huang X. A Review of Battery Fires in Electric Vehicles. Fire Technol. 2020, 56, 1361–1410. 10.1007/s10694-019-00944-3.

[ref4] Goikolea E.; Palomares V.; Wang S.; de Larramendi I. R.; Guo X.; Wang G.; Rojo T. Na-ion batteries—approaching old and new challenges. Adv. Energy Mater. 2020, 10, 2002055 10.1002/aenm.202002055.

[ref5] Kuhn A.; Kunze M.; Sreeraj P.; Wiemhöfer H. D.; Thangadurai V.; Wilkening M.; Heitjans P. NMR Relaxometry as a Versatile Tool to Study Li Ion Dynamics in Potential Battery Materials. Solid State Nucl. Magn. Reson. 2012, 42, 2–8. 10.1016/j.ssnmr.2012.02.001. 22364761

[ref6] Epp V.; Nakhal S.; Lerch M.; Wilkening M. Two-Dimensional Diffusion in Li0.7NbS2 as Directly Probed by Frequency-Dependent 7Li NMR. J. Phys. Condens. Matter 2013, 25, 195402 10.1088/0953-8984/25/19/195402. 23604197

[ref7] Wilkening M.; Heitjans P. From Micro to Macro: Acess to Long Range Li+ Diffusion Parameters in Solids via Microscopic 6,7Li Spin Alignment Echo Nmr Spectroscopy. Phys. Chem. Chem. Phys. 2012, 13, 53–65. 10.7208/chicago/9780226317298.003.0007. 21954143

[ref8] Kuhn A.; Epp V.; Schmidt G.; Narayanan S.; Thangadurai V.; Wilkening M. Spin-Alignment Echo NMR: Probing Li + Hopping Motion in the Solid Electrolyte Li 7La 3Zr 2O 12 with Garnet-Type Tetragonal Structure. J. Phys. Condens. Matter 2012, 24, 035901 10.1088/0953-8984/24/3/035901. 22179497

[ref9] Heitjans P.; Schirmer A.; Indris S. NMR and β-NMR Studies of Diffusion in Interface-Dominated and Disordered Solids. Diffus. Condens. Matter 2005, 367–415. 10.1007/3-540-30970-5_9.

[ref10] Müller K.; Geppi M. Solid State NMR: Principles, Methods, and Applications; John Wiley & Sons, 2021.

[ref11] Bloembergen N.; Purcell E. M.; Pound R. V. Relaxation Effects in Nuclear Magnetic Resonance Absorption. Phys. Rev. 1948, 73, 679–712. 10.1103/PhysRev.73.679.

[ref12] Richards P. M. Effect of Low Dimensionality on Prefactor Anomalies in Superionic Conductors. Solid State Commun. 1978, 25, 1019–1021. 10.1016/0038-1098(78)90896-7.

[ref13] Epp V.; Wilkening M. Fast Li Diffusion in Crystalline LiBH4 Due to Reduced Dimensionality: Frequency-Dependent NMR Spectroscopy. Phys. Rev. B 2010, 82, 020301 10.1103/PhysRevB.82.020301.

[ref14] Wilkening M.; Heitjans P. Li Jump Process in H- Li0.7 Ti S2 Studied by Two-Time Li7 Spin-Alignment Echo NMR and Comparison with Results on Two-Dimensional Diffusion from Nuclear Magnetic Relaxation. Phys. Rev. B: Condens. Matter Mater. Phys. 2008, 77, 1–13. 10.1103/PhysRevB.77.024311.

[ref15] Gombotz M.; Lunghammer S.; Breuer S.; Hanzu I.; Preishuber-Pflügl F.; Wilkening H. M. R. Spatial Confinement-Rapid 2D F- Diffusion in Micro- and Nanocrystalline RbSn2F5. Phys. Chem. Chem. Phys. 2019, 21, 1872–1883. 10.1039/c8cp07206j. 30632556

[ref16] Delmas C.; Fouassier C.; Hagenmuller P. Structural Classification and Properties of Layered Oxides. Phys. B+C 1980, 99, 81–85. 10.1016/0378-4363(80)90214-4.

[ref17] Weller M.; Sacchetti A.; Ott H. R.; Mattenberger K.; Batlogg B. Melting of the Na Layers in Solid Na0.8CoO2. Phys. Rev. Lett. 2009, 102, 6–9. 10.1103/PhysRevLett.102.056401. 19257527

[ref18] Villa M.; Bjorkstam J. L. Na23 and Al27 in β-Alumina Solid Electrolytes. Phys. Rev. B 1980, 22, 5033–5042. 10.1103/PhysRevB.22.5033.

[ref19] Carlier D.; Blangero M.; Ménétrier M.; Pollet M.; Doumerc J. P.; Delmas C. Sodium Ion Mobility in NaxCoO2 (0.6 <× <0.75) Cobaltites Studied by 23Na MAS NMR. Inorg. Chem. 2009, 48, 7018–7025. 10.1021/ic900026c. 19419150

[ref20] Han O. H.; Jung J. K.; Yi M. Y.; Kwak J. H.; Shin Y. J. Sodium Ion Dynamics in the Nonstoichiometric Layer-Type Oxide Na0.67Ni0.33Ti0.67O2 Studied by 23Na NMR. Solid State Commun. 2000, 117, 65–68. 10.1016/S0038-1098(00)00431-2.

[ref21] Smirnova O. A.; Rocha J.; Nalbandyan V. B.; Kharton V. V.; Marques F. M. B. Crystal Structure, Local Sodium Environments and Ion Dynamics in Na0.8Ni0.6Sb0.4O2, a New Mixed Antimonate. Solid State Ionics 2007, 178, 1360–1365. 10.1016/j.ssi.2007.08.002.

[ref22] Evstigneeva M. A.; Nalbandyan V. B.; Petrenko A. A.; Medvedev B. S.; Kataev A. A. A New Family of Fast Sodium Ion Conductors: Na2M 2TeO6 (M = Ni, Co, Zn, Mg). Chem. Mater. 2011, 23, 1174–1181. 10.1021/cm102629g.

[ref23] Schmidt W.; Berthelot R.; Sleight A. W.; Subramanian M. A. Solid Solution Studies of Layered Honeycomb-Ordered Phases O 3 – Na 3 M 2 SbO 6 (M 1/4 Cu, Mg, Ni, Zn). J. Solid State Chem. 2013, 201, 178–185. 10.1016/j.jssc.2013.02.035.

[ref24] Li X.; Bianchini F.; Wind J.; Pettersen C.; Wragg D. S.; Vajeeston P.; Fjellvåg H. Insights into Crystal Structure and Diffusion of Biphasic Na 2 Zn 2 TeO 6. ACS Appl. Mater. Interfaces 2020, 12, 28188–28198. 10.1021/acsami.0c05863. 32484658 PMC7467548

[ref25] Bianchini F.; Fjellvåg H.; Vajeeston P. Nonhexagonal Na Sublattice Reconstruction in the Super-Ionic Conductor Na 2 Zn 2 TeO 6: Insights from Ab Initio Molecular Dynamics. J. Phys. Chem. C 2019, 123, 4654–4663. 10.1021/acs.jpcc.8b10362.

[ref26] Li Y.; Deng Z.; Peng J.; Chen E.; Yu Y.; Li X.; Luo J.; Huang Y.; Zhu J.; Fang C.; Li Q.; Han J.; Huang Y. A P2-Type Layered Superionic Conductor Ga-Doped Na2Zn2TeO6for All-Solid-State Sodium-Ion Batteries. Chem. - Eur. J. 2018, 24, 1057–1061. 10.1002/chem.201705466. 29226609

[ref27] Wu J. F.; Wang Q.; Guo X. Sodium-Ion Conduction in Na2Zn2TeO6 Solid Electrolytes. J. Power Sources 2018, 402, 513–518. 10.1016/j.jpowsour.2018.09.048.

[ref28] Deng Z.; Gu J.; Li Y.; Li S.; Peng J.; Li X.; Luo J.; Huang Y.; Fang C.; Li Q.; Han J.; Huang Y.; Zhao Y. Ca-Doped Na 2 Zn 2 TeO 6 Layered Sodium Conductor for All-Solid-State Sodium-Ion Batteries. Electrochim. Acta 2019, 298, 121–126. 10.1016/j.electacta.2018.12.092.

[ref29] Sau K.; Kumar P. P. Ion Transport in Na2M2TeO6: Insights from Molecular Dynamics Simulation. J. Phys. Chem. C 2015, 119, 1651–1658. 10.1021/jp5094349.

[ref30] Sau K.; Kumar P. P. Role of Ion-Ion Correlations on Fast Ion Transport: Molecular Dynamics Simulation of Na2Ni2TeO6. J. Phys. Chem. C 2015, 119, 18030–18037. 10.1021/acs.jpcc.5b04087.

[ref31] Sau K. Influence of Ion–Ion Correlation on Na+ Transport in Na2Ni2TeO6: Molecular Dynamics Study. Ionics 2016, 22, 2379–2385. 10.1007/s11581-016-1782-2.

[ref32] Hempel F. S.; Bianchini F.; Arstad B.; Fjellvåg H. Effects of Ga Substitution on the Local Structure of Na2Zn2TeO6. Inorg. Chem. 2022, 61, 13067–13076. 10.1021/acs.inorgchem.2c01431. 35944025 PMC9400102

[ref33] Coelho A. A. TOPAS and TOPAS-Academic: An Optimization Program Integrating Computer Algebra and Crystallographic Objects Written in C++: An. J. Appl. Crystallogr. 2018, 51, 210–218. 10.1107/S1600576718000183.

[ref34] Thurber K. R.; Tycko R. Measurement of Sample Temperatures under Magic-Angle Spinning from the Chemical Shift and Spin-Lattice Relaxation Rate of 79Br in KBr Powder. J. Magn. Reson. 2009, 196, 84–87. 10.1016/j.jmr.2008.09.019. 18930418 PMC2632797

[ref35] Massiot D.; Fayon F.; Capron M.; King I.; Le Calvé S.; Alonso B.; Durand J. O.; Bujoli B.; Gan Z.; Hoatson G. Modelling One- and Two-Dimensional Solid-State NMR Spectra. Magn. Reson. Chem. 2002, 40, 70–76. 10.1002/mrc.984.

[ref36] Kresse G.; Hafner J. Ab Initio Molecular Dynamics for Liquid Metals. Phys. Rev. B 1993, 47, 558–561. 10.1103/PhysRevB.47.558. 10004490

[ref37] Kresse G.; Furthmüller J. Efficiency of Ab-Initio Total Energy Calculations for Metals and Semiconductors Using a Plane-Wave Basis Set. Comput. Mater. Sci. 1996, 6, 15–50. 10.1016/0927-0256(96)00008-0. 9984901

[ref38] Kresse G.; Furthmüller J. Efficient Iterative Schemes for Ab Initio Total-Energy Calculations Using a Plane-Wave Basis Set. Phys. Rev. B 1996, 54, 11169–11186. 10.1103/PhysRevB.54.11169. 9984901

[ref39] Kresse G.; Joubert D. From Ultrasoft Pseudopotentials to the Projector Augmented-Wave Method. Phys. Rev. B 1999, 59, 1758–1775. 10.1103/PhysRevB.59.1758.

[ref40] Li X.; Bianchini F.; Wind J.; Vajeeston P.; Wragg D.; Fjellvåg H. P2 Type Layered Solid-State Electrolyte Na 2 Zn 2 TeO 6: Crystal Structure and Stacking Faults. J. Electrochem. Soc. 2019, 166, A3830–A3837. 10.1149/2.1231915jes.

[ref41] Nosé S. Constant Temperature Molecular Dynamics Methods Limitations in Simulations in the Microcanonical Ensemble. Prog. Theor. Phys. Suppl. 1991, 103, 1–46. 10.1143/PTPS.103.1.

[ref42] Nosé S. A Unified Formulation of the Constant Temperature Molecular Dynamics Methods. J. Chem. Phys. 1984, 81, 511–519. 10.1063/1.447334.

[ref43] Bylander D. M.; Kleinman L. Energy Fluctuations Induced by the Nose Thermostat. Phys. Rev. B 1992, 46, 13756 10.1103/PhysRevB.46.13756. 10003434

[ref44] Goret G.; Aoun B.; Pellegrini E. MDANSE: An Interactive Analysis Environment for Molecular Dynamics Simulations. J. Chem. Inf. Model. 2017, 57, 1–5. 10.1021/acs.jcim.6b00571. 28026944

[ref45] Larsen A. H.; Mortensen J. J.; Blomqvist J.; Castelli I. E.; Christensen R.; Dułak M.; Friis J.; Groves M. N.; Hammer B.; Hargus C.; Hermes E. D.; Jennings P. C.; Jensen P. B.; Kermode J.; Kitchin J. R.; Kolsbjerg E. L.; Kubal J.; Kaasbjerg K.; Lysgaard S.; Maronsson J. B.; Maxson T.; Olsen T.; Pastewka L.; Peterson A.; Rostgaard C.; Schiøtz J.; Schütt O.; Strange M.; Thygesen K. S.; Vegge T.; Vilhelmsen L.; Walter M.; Zeng Z.; Jacobsen K. W. The Atomic Simulation Environment-a Python Library for Working with Atoms. J. Phys. Condens. Matter 2017, 29, 273002 10.1088/1361-648X/aa680e. 28323250

[ref46] Bahn S. R.; Jacobsen K. W. An Object-Oriented Scripting Interface to a Legacy Electronic Structure Code. Comput. Sci. Eng. 2002, 4, 56–66. 10.1109/5992.998641.

[ref47] QUIP. https://libatoms.github.io/QUIP/ (accessed April 18, 2023).

[ref48] Momma K.; Izumi F. VESTA: A Three-Dimensional Visualization System for Electronic and Structural Analysis. J. Appl. Crystallogr. 2008, 41, 653–658.

[ref49] d’Espinose de Lacaillerie J. B.; Fretigny C.; Massiot D. MAS NMR Spectra of Quadrupolar Nuclei in Disordered Solids: The Czjzek Model. J. Magn. Reson. 2008, 192, 244–251. 10.1016/j.jmr.2008.03.001. 18362082

