# Supplementary material for: Dynamics of Interlayer Na-Ions in Ga-Substituted Na2Zn2TeO6 (NZTO) Studied by Variable-Temperature Solid-State 23Na NMR Spectroscopy and DFT Modeling
Source: ACS Phys Chem Au. 2023 May 4;3(4):394–405. doi: 10.1021/acsphyschemau.3c00012 (PMC10375874; doi:10.1021/acsphyschemau.3c00012)
Supplement: Supplementary file 1 — pg3c00012_si_001.pdf [file pg3c00012_si_001.pdf]

Supplementary information

Dynamics of Interlayer Na-ions in Ga-substituted $Na_2Zn_2TeO_6$ (NZTO) studied by variable temperature solid state ^{23}Na NMR spectroscopy and DFT modeling.

Frida Sveen Hempel [1,2,#], *Charlotte Martineau-Corcos* [3], *Federico Bianchini* [2,†], *Helmer Fjellvåg* [2] *and Bjørnar Arstad* [1,*]

[1] SINTEF Industry, Forskningsveien 1, 0373 Oslo, Norway

[2] Department of Chemistry and Center for Materials Science and Nanotechnology, University of Oslo, Oslo 0371, Norway

[3] CortecNet, 7 avenue du Hoggar, 91940 Les Ulis, France

Present address:

Morrow Technologies AS, c/o Institute for Energy Technology, Instituttveien 18, 2007 Kjeller, Norway

† Center for Bioinformatics, University of Oslo Gaustadalléen 30 N-0373 Oslo, Norway

* Corresponding author: Bjørnar Arstad

S1 Synthesis of materials

The materials in this work are the same as we have previously published.[1] The synthesis procedure and XRD plots are reported here for completeness.

Precursors ZnO (Sigma Aldrich, 99.99%), TeO_2 (Sigma Aldrich, 99.995%) and $Ga(NO_3)_3$ * $x$$H_2O$ (Sigma Aldrich, 99.9%) were added in stoichiometric ratio, and Na_2CO_3 (Sigma Aldrich, >99.5%) with a 10% excess to account for evaporation. The ZnO, Na_2CO_3 and TeO_2 is dissolved in nitric acid (Sigma Aldrich, 65%) in a magnetic stirrer on a hot plate at 50°C until solution becomes transparent. Subsequently, $Ga(NO_3)_3$ * $x$$H_2O$ is dissolved in water and added. When everything is mixed, citric acid (Sigma Aldrich, 99.5%) compound is added in a ratio of 5:1 to the cations in the precursors, before the solution is heated to 180°C. After the NO_x gas to evaporated and a gel is formed, it was left overnight at 180°C. The powder was then heated to 450°C for 12h, before ball milling at 600 rpm for 20 min. It was then sintered at at 900°C for samples x = 0.00-0.10 and 800°C for x = 0.15-0.20 for 3h, with a heating/cooling rate of 5°C. The compound is pressed into pellets and covered at the top and bottom the mother powder, to limit evaporation and reduce diffusion length.

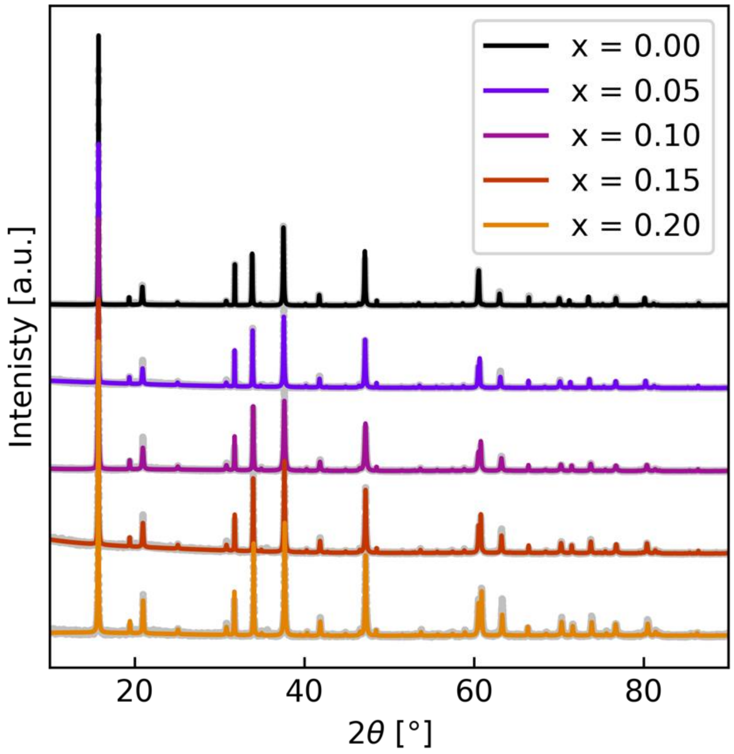

S2 Water estimation

The amount water in the materials was estimated by the following method and is based on carefully executed NMR experiments to obtain quantitative data. We further assume that we can compare absolute integral values from sample to sample (from one NMR experiment to another NMR experiment) when the following protocol is applied. A 50 µl volume (HR-MAS) rotor was carefully washed in acetone and dried before use. Several potential compounds were tried for use as reference for quantitative measurements of levels of H atoms, but most had strong H couplings and integrations that were ambiguous. However, one compound that suited our work was HNa_2PO_4 (Sigma-Aldrich 99.99%, water free). Before use some of the powder was dispersed on a glass plate and dried overnight at 150 C ° to remove water contamination. A carefully weighted amount of HNa_2PO_4 (0.0155 g) was then packed in the HR-MAS rotor and inserted into the NMR probe. Sample handling/packing in an Ar-glove box and rapid packing in air were carried out and compared and it was not found any significant differences, however, a minor component of water was left in the reference that could not be removed at moderate treatment. 0.0155 g is large enough to give reasonable low integration error and we do not need to subtract the background but is also small enough to ensure that all the powder is well within the volume of the coil in the stator. We compared with an external coil + stator to ensure this. Powder outside the coil could give contributions but at non reproducible ways as the pulse angle is smaller for powders outside the coil volume. The amount water in the reference was about 0.4% of the main H peak. For all experiments the exact same experimental setup was used, included the same X-channel tuning, receiver gain and others. A pre-run was carried out before each experiment before a second tune and match was carried out. Acquisition times were tested, and we are quite sure we have used a suited time for signal/FID acquisition, neither too short not too long. The NMR parameter setup was optimized ahead in similar experiments to find suited acquisitions parameters, that later were not changed. The number of scans in all experiments was 40. The recycle delay was for all samples was optimized and checked by doing a series of experiment with increasing recycle delay. For all NZTO based samples reported in this article it was enough to use a 10 s recycle delay, but all averaged numbers are based on experiments with recycle delays from 5 s to 30 s. For the reference HNa_2PO_4 we had several runs to find a suited recycle delay. In the end we used 6000 seconds (2 days and ~18-hour total instrument time) between each scan. It was possible to observe that a recycle delay of 10000 seconds might give a slightly larger area, but we judged it small enough that the underestimation of the area of H in HNa_2PO_4 should be marginally and insignificant compared to errors in the integration of the total areas in the NZTO based

samples. Areas of spinning sidebands were included in the final integrated values. Zero filling and baseline corrections were carried out before integration. However, the baseline corrections were not trivial and several attempts of baseline correction + integrations for each sample were carried out. Since we used five different experiments, each with different recycle delay, for each estimated number we could average five different baseline corrections + integrations from five different NMR experiments on the same sample. By comparing the area from the H atom in the reference sample and through some Excel sheet calculation an equivalent water amount could be estimated for the reference, and this could then be compared to the integrated level of water in the NZTO based samples.

S3 Additional information on ^{23}Na 100-293K NMR

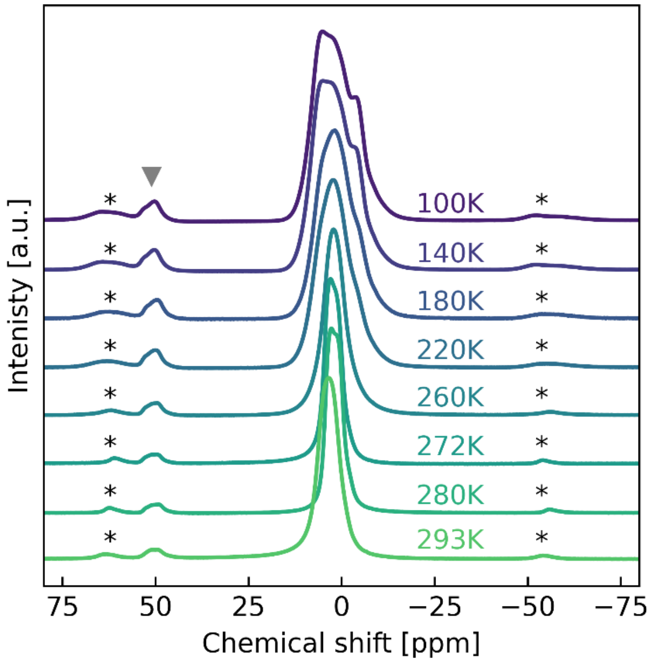

Figure S1: Wider spectral range of the ^{23}Na NMR spectra for NZTO from 100 K to 293 K at 18.8T. Spinning sidebands marked with * and 45ppm feature denoted with a grey triangle.

S4 Simulation parameters for Czjzek distribution

Table S1: Simulation parameters for Czjzek distribution shown in figure 3.

Peak	Position [Hz]	Amplitude [10^8]	FWHM CS	CQ [kHz]	Integrated intensity [%]	Assigned prism
Left	6.6	9.17	2.85	1789	52	*6g*
Middle	1.4	8.38	5.23	1608	39	*4f*
Right	-4.1	3.16	1.41	1265	9	*2a*

S5 Simulations of a three-site exchange system

There are many assumptions to consider in the application of the calculated spectra below, and they will be discussed in some detail here. The calculations are not intended to be a curve fitting giving a "correct" model of the materials in study, but to provide some background for discussing the complex situation, and for providing some visual examples. A standard matrix representation of a three-site exchange system is used, and final signals are generated by multiplication with a decaying exponential function before Fourier transforming the total sum of signals (3) for the spectra shown below.

The first spectrum is designed to be qualitative like the one observed during MAS at 100 K. The peak shapes are Lorentzian and at 100 K this is not the best approximation but at higher temperatures with more Na dynamics this assumption is not that bad as shown by the RT MAS 18.8 T ^{23}Na spectrum in Figure S2 , section S6, further below. Peak shape narrowing due to reduced quadrupolar-, and dipole-dipole -interactions may be simulated by adjusting a line broadening factor (T1#'s) in the calculations. Changes in peak positions due to a reduction of the magnitude of the 2nd order quadrupolar Hamiltonian which is again due to ion dynamics may be simulated by a small shift of the peak's center of gravity towards left, possibly also together with a narrower peak through an adjusted line broadening factor. The following panels of simulated spectra, and with explanation texts, are intended to provide indications the general processes that might take place during heating the samples. The model frequencies are relative to 0 in the spectra. K's are jump rates, and T1's are model parameters for linebroadening.

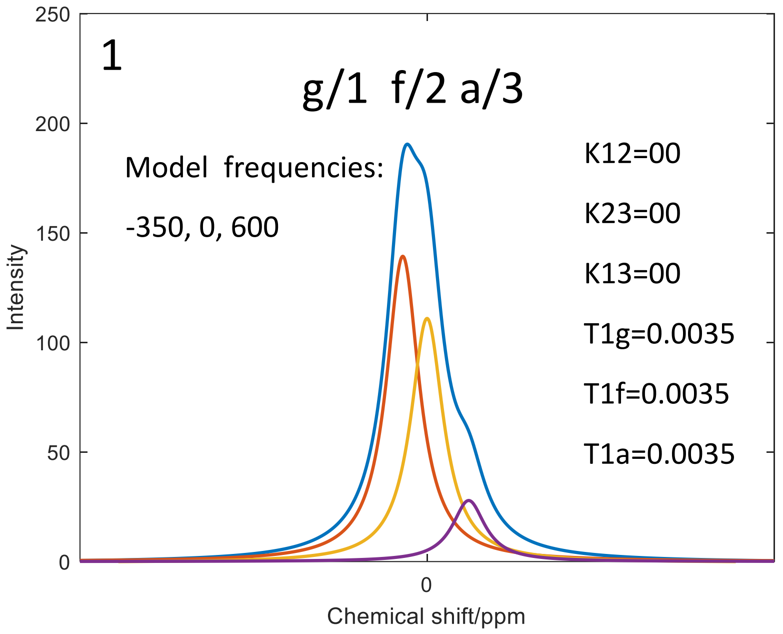

Panel 1: Starting configuration. g/1 f/2 a/3 indicates positions and id (both letter and number). K's and T1's are model parameters simulating jump rates between positions (e.g., K12 is jump rate between 1 and 2) and linebroadening, respectively. The peaks become narrower with an increase in the T1 values. Simulated positions of peaks 1, 2, and 3 are at relative model frequencies of -350, 0, and 600 Hz, respectively. These frequencies are model parameters and not the measured distance between peaks in the data acquired at 18.8 T.

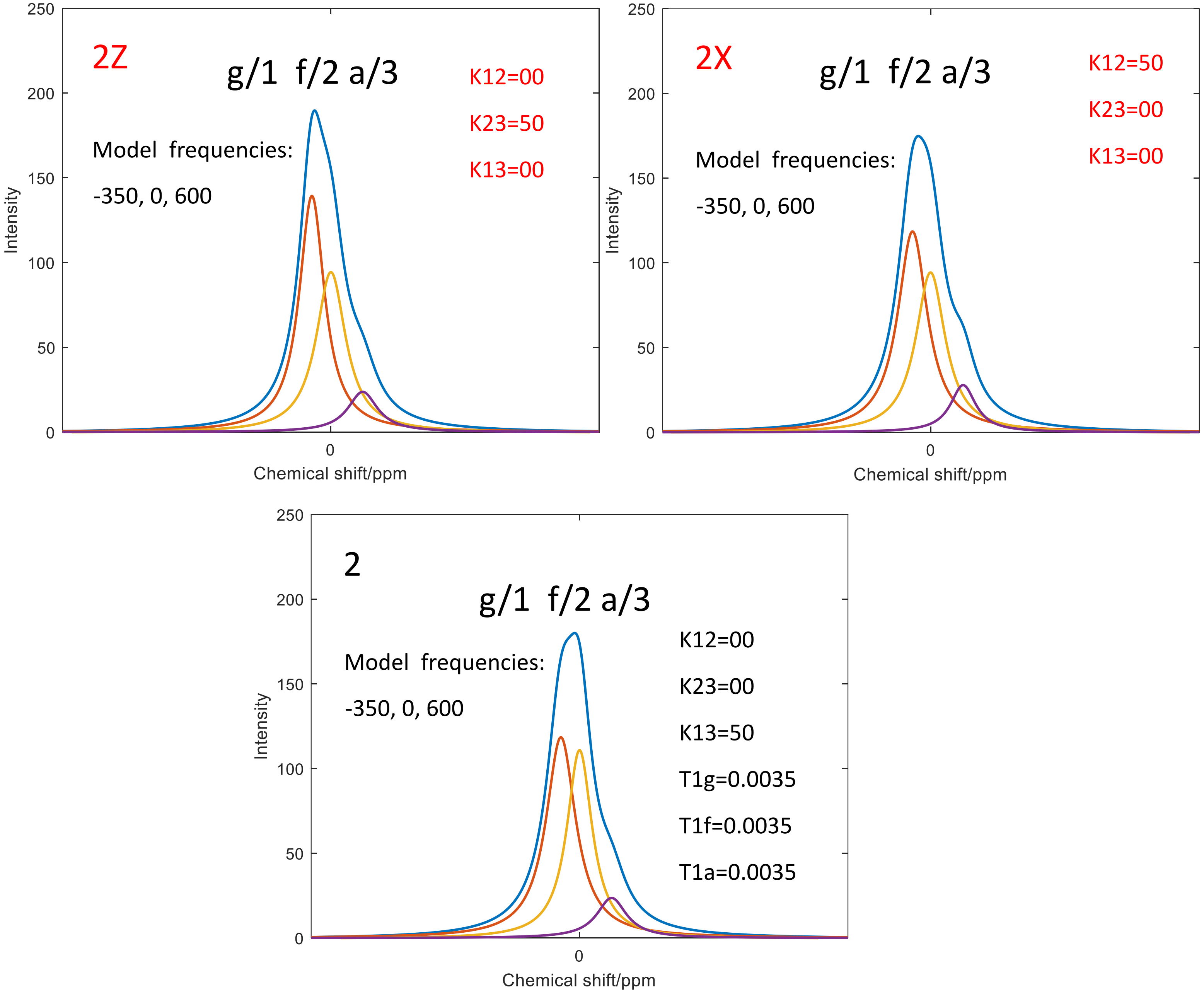

Panel 2, 2X, 2Z. We see that an increase in K13 instead of K12 or K23 give a spectrum that resembles measured data much better, which is also in line with previous reports. The tendency of the spectra in Figure 3 in the manuscript is to have the highest intensity in the middle of the total peak shape. This controls what sites the initial exchange is going between.

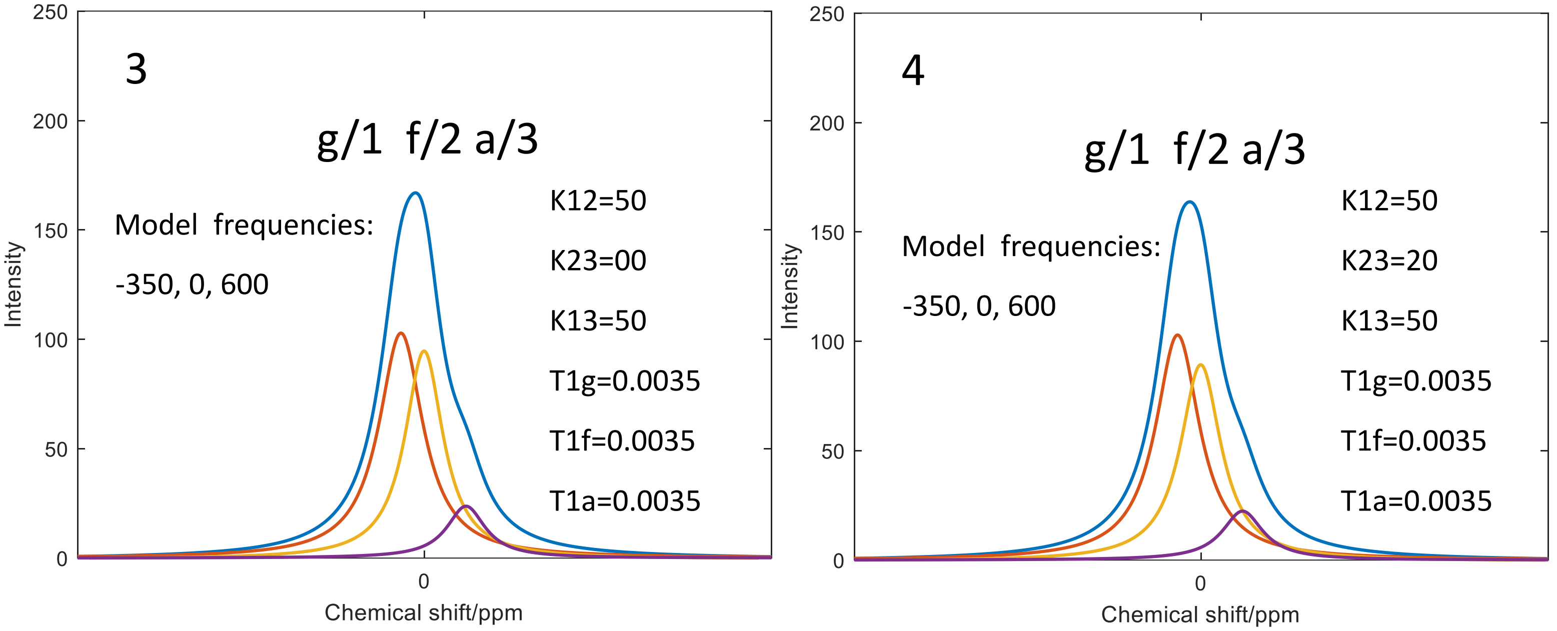

Panel 3 and 4 are spectra from a further increase in jump rates between the positions. In 4 all three sites are experiencing intermixing of ions. Panel 4 is looking somewhat like the 260 K spectrum in Figure 3. However, to resemble the 272 and 280 K spectra we had to adjust differently than just increasing jump rates.

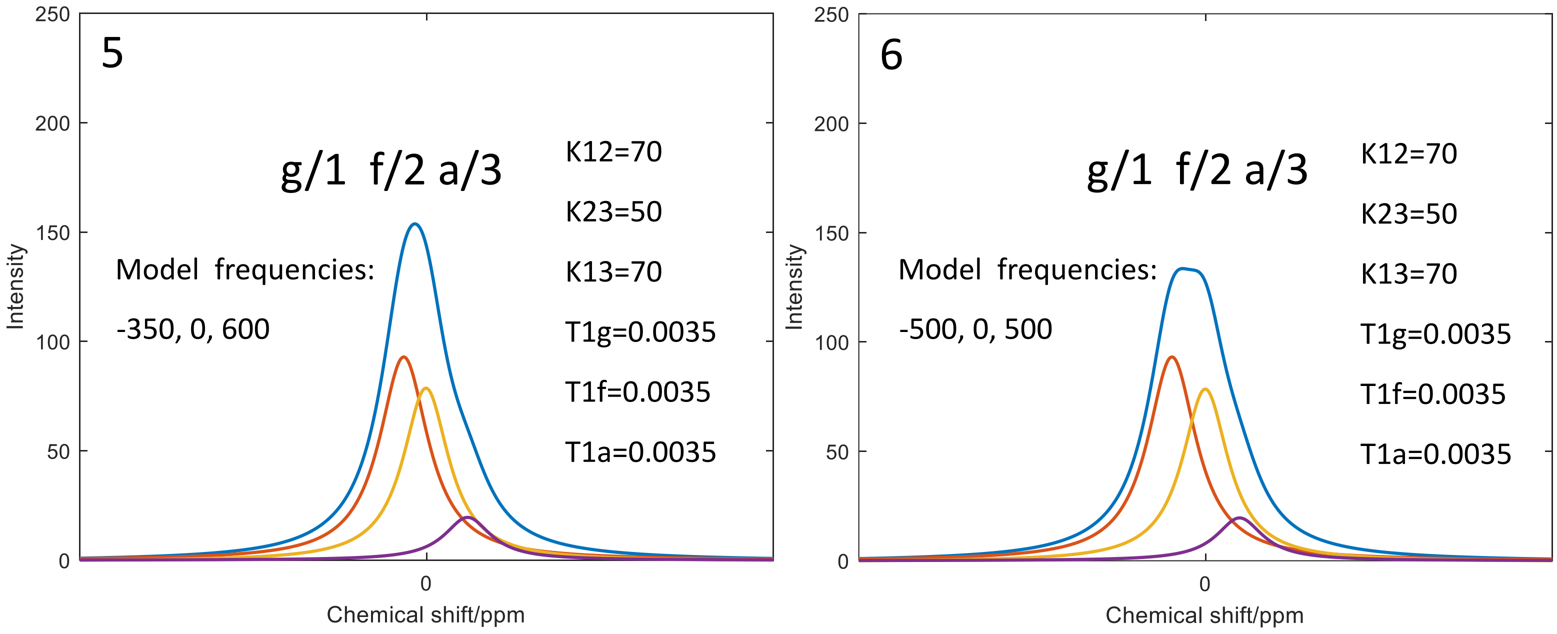

Panel 5 shows a further increase in jump rates relative to panel 4. Panel 6 has the same jump rates as shown in panel 5, but a small change in the positions of peak 1 and 3 to the left to simulate reduced quadrupolar couplings. Simulated positions of peaks 1, 2, and 3 in panel 6 are at relative model frequencies -500, 0, and 500 Hz respectively, in contrast to -350, 0, and 600 Hz for the panel 1-5 simulations.

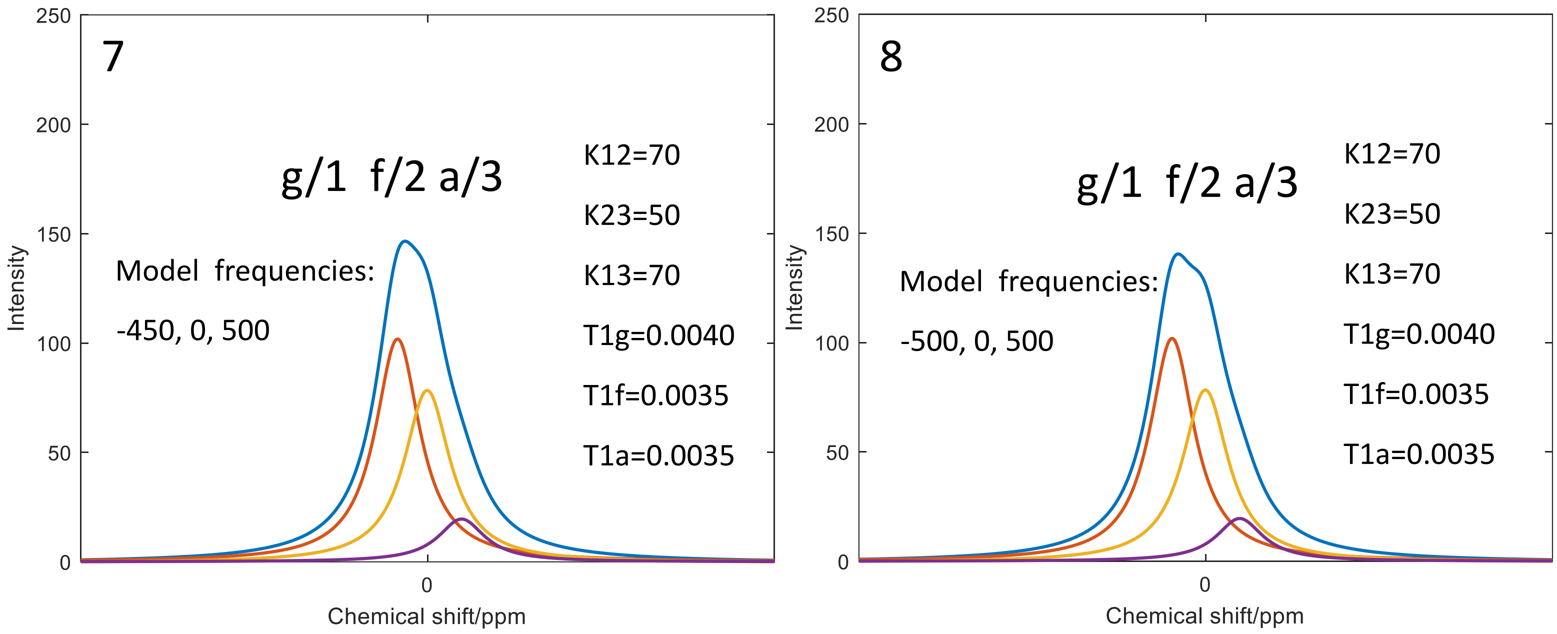

Panel 7 is similar to panel 6 except a slightly narrowing of peak 1 simulated by a higher peak broadening value but with lesser shift in peak positions as the simulated positions of peaks 1, 2, and 3 in panel 7 are at relative model frequencies of -450, 0, and 500 Hz respectively. Panel 8 is the same as 7 except that the peak positions are back to where the peaks in panel 6 were, i.e., the simulated positions of peaks 1, 2, and 3 in panel 8 are at relative model frequencies of -500, 0, and 500 Hz respectively. Panel 8's peak 1 is narrower compared to in panel 6. Panel 8 gives a better similarity with the 272 and 280 K experimental data compared to panel 5, 6, and 7. To approach the 293 K spectrum with a rather symmetric peak we did the simulations shown in panel 9 and 10.

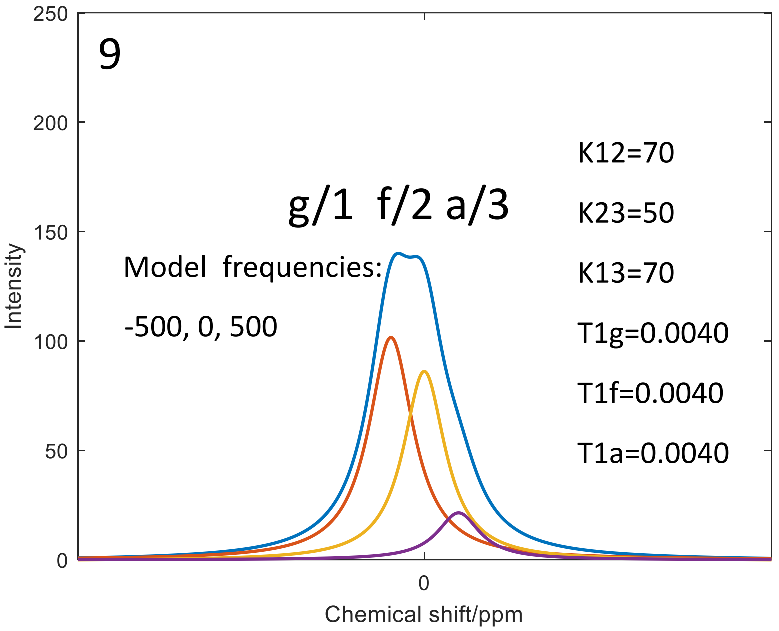

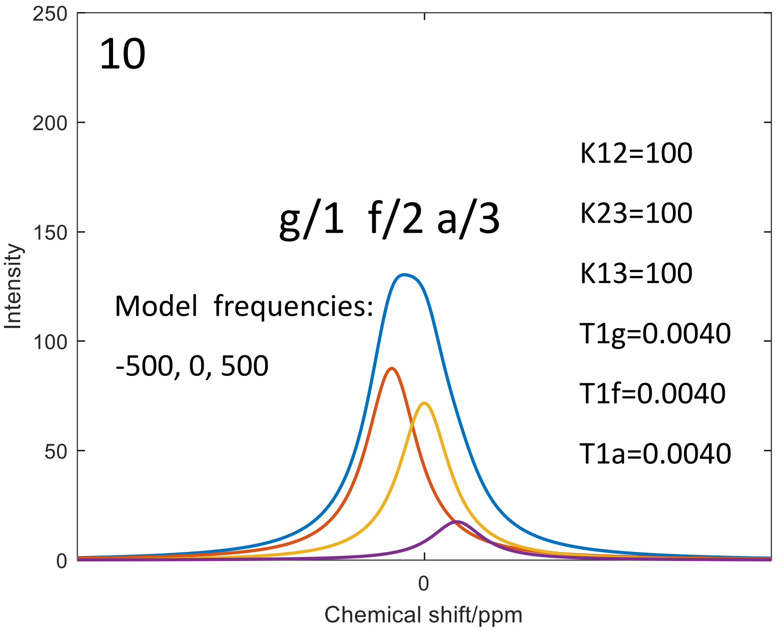

Panel 9 have the same peak positions and jump rates as 8 (model frequencies of -500, 0, and 500 Hz) but peak 2 and 3 are now narrower, and now equal to peak 1. An even further increase in jump rates lead to the spectrum shown in panel 10 and is the spectrum that most resembles the 293 K spectrum.

The overall sequence describing best the measured spectra is then 1 > 2 > 4 > 6 > 8 >9 > 10.

S6 Line fitting of the MAS spectrum acquired at 18.8 T

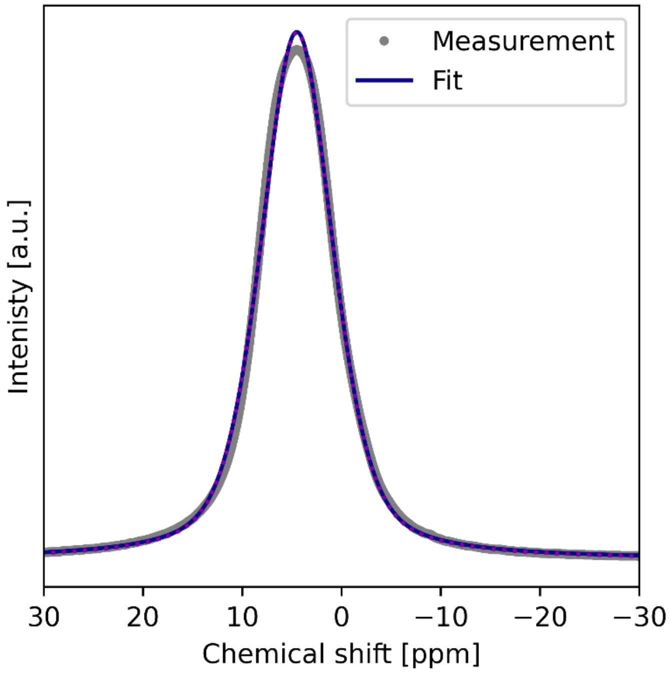

Figure S2: Curve fitting of the ^{23}Na spectrum at 293 K at 18.8 T. the main peak is basically symmetric as seen by curve fitting with a Gaussian and/or a Lorentzian function. A single component is clearly not sufficient for a perfect description, but the high symmetry of the peak demonstrates that the field is strong enough to reduce the quadropolar coupling to a very low value, which would otherwise give a peak with a tailing to the right or some sort of unsymmetric shape.

S7 Stacked plot of Ga-doped materials $Na_{2-x}Zn_{2-x}Ga_xTeO_6$ (x = 0.05, 0.10, 0.15, 0.20) from 100 K up to 293 K at 18.8 T.

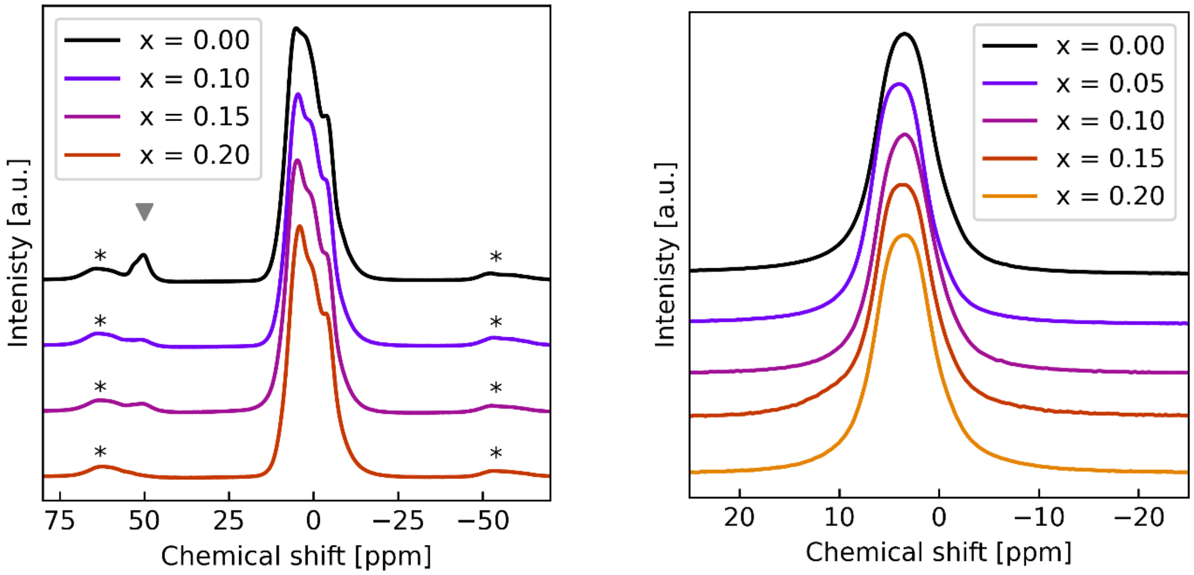

Figure S3: ^{23}Na MAS NMR at 18.8T with MAS rate 12.5 kHz for Ga-substituted samples at (a) 100K for all samples except x = 0.05 and (b) room temperature for all samples.

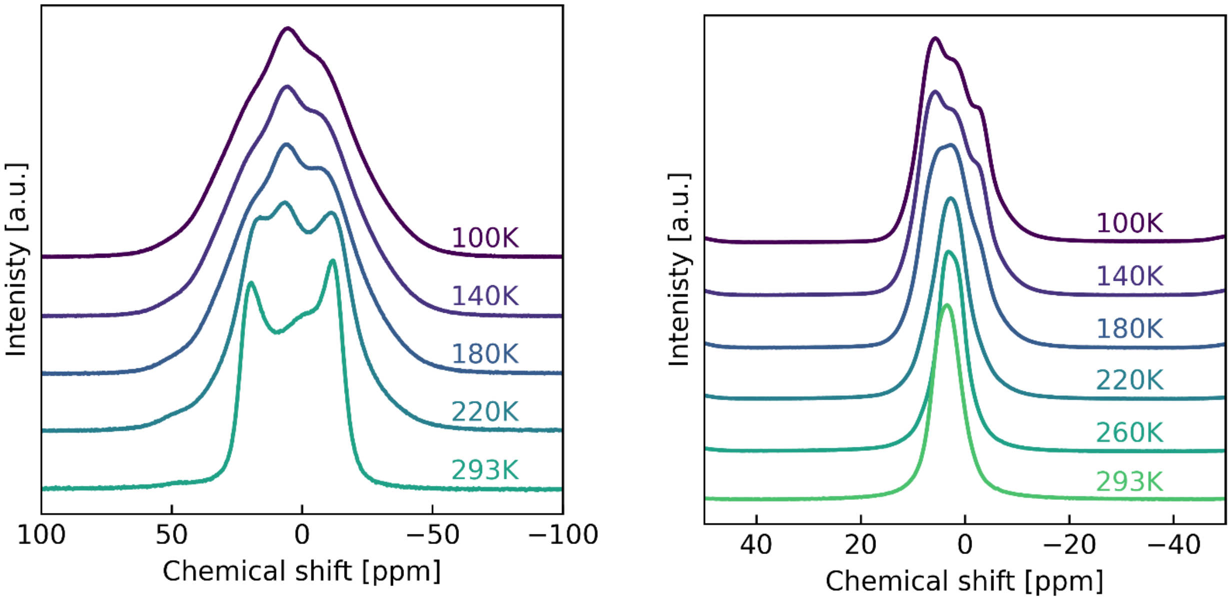

Figure S4: NMR spectra of x = 0.10 from 100 K to 293 K in a 18.8 T field. (a) static and (b) MAS with rate 12.5 kHz.

S8 Relaxation measurements

Topspin 3.6.2 was used to analyse the relaxation data, and the T_1 curve fit for values found from the "Saturation Recovery" method is principally described by the satrec (Bruker defined name) function:

$$Mz = Mz_0 - Mz_0 * \exp\left(\left({}^{\tau}/_{T_1}\right)^{\gamma}\right)$$

Mz is the magnetization along the magnetic field and Mz_0 is the equilibrium magnetization after fully recovery along the magnetic field. γ is a stretching factor. However, the uxnmrt1 (Bruker defined name) function is often employed instead, as this allows for experimental errors like imperfect saturation and background variations giving a remnant ^{23}Na signal at t=0. This unction is given by:

$$Mz = Mz_0 + P\exp\left(\left({}^{\tau}/_{T_1}\right)^{\gamma}\right)$$

P is a factor correcting residual peak intensities and other systematic variations/imperfections. Repeated measurements at a decided temperature gave variations in T1 up to about 10 µs, which is well within the difference between most points. Upon remeasuring on different days, some of the difference in T_1 could be up to 40 µs in a single measurement, but an average difference between 5 and 20 µs were typically observed. One reason may be that even with apparent stable temperatures during measurement there might be some variations due to variable temperatures within the sample powder. We therefore decided to reduce the uncertainty in T_1 values at each temperature by using an average value from several measurements and find that the trends in measured T_1 values could be trusted.

Table S2: Average measured T_1 values (µs) for all samples x = 0.00, 0.05, 0.10, 0.15 and 0.20 measured at 11.7 T.

T [C]	T [K]	1000/T	NZTO	NZGTO 0.05	NZGTO 0.1	NZGTO 0.15	NZGTO 0.2
22	295	3.39	500	586	553	484	446
40	313	3.19	424	511	496	423	407
60	333	3.00	359	430	437	385	390
80	353	2.83	316	380	396	363	382
100	373	2.68	304	342	367	362	393
120	393	2.54	291	321	364	377	432
140	413	2.42	294	316	369	400	483
160	433	2.31	319	320	388	441	537
180	453	2.21	355	359	431	506	590
200	473	2.11	419	419	500	596	687
215	488	2.05	-	499	567	691	769
220	493	2.03	512	-	-	-	-

230	503	1.99	-	609	637	772	839
235	508	1.97	648	-	-	-	-

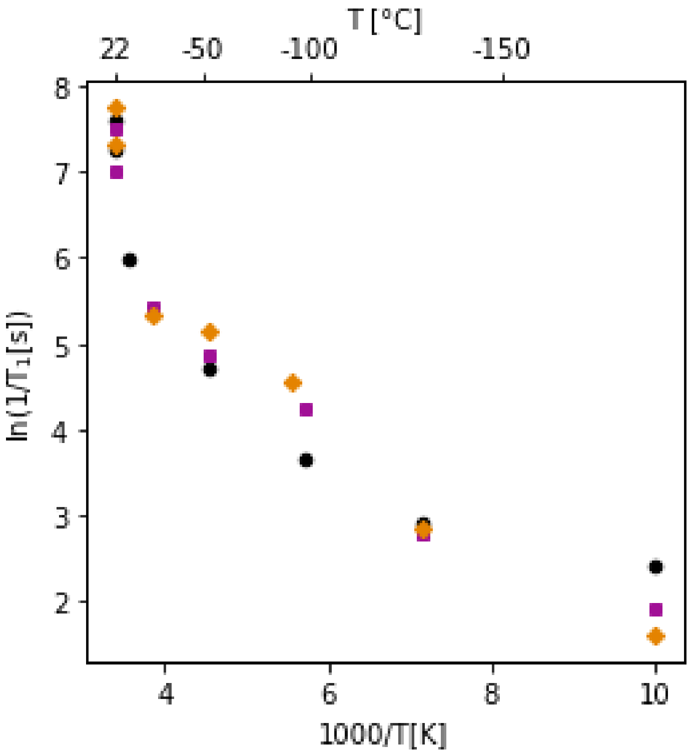

Figure S5. T_1 relaxation constants from 100 K up to 293 K measured at 18.8 T. Note the much larger T_1 value at 100 K compared to at 293 K.

S9 DFT calculation of Na mobility

The mean square displacement of Na atoms is computed as:

$$MSD = \langle \, | \, \mathbf{x}(t) \, - \mathbf{x}_0|^2 \, \rangle = \frac{1}{N}\sum_{i=1}^{N}\left| \, \mathbf{x}^{(\mathrm{i})}(t) \, - \mathbf{x}^{(\mathrm{i})}(0)\right|^2$$

where $x^{(i)}(t)$ is the position of the ith Na atom at time *t*, *N* the total number of Na atoms.

The MSD is related to the diffusion coefficient by the MSD(*t*)= 2nDt + C relation, where *n* is the dimensionality of the diffusion channels, *D* the diffusion coefficient and *C* a constant representing the vibrational contribution to the MDS. After verifying the absence of diffusion along *z*, *n* has been fixed to 2. When the diffusion coefficient is obtained as a function of temperature, the Arrhenius law is used to compute the activation energy and to evaluate the diffusion coefficient at room temperature. Finally, the mobility is obtained from the diffusion coefficient using the Nernst-Einstein relation.

$$\sigma = \frac{DNq^2}{k_B T}$$
